# Accommodating unobservability to control flight attitude with optic flow

**DOI:** 10.1038/s41586-022-05182-2

**Published:** 2022-10-19

**Authors:** Guido C. H. E. de Croon, Julien J. G. Dupeyroux, Christophe De Wagter, Abhishek Chatterjee, Diana A. Olejnik, Franck Ruffier

**Affiliations:** 1 Micro Air Vehicle Laboratory, Control and Simulation, Faculty of Aerospace Engineering, Delft University of Technology, Delft, the Netherlands; 2 Aix Marseille Université, CNRS, ISM, Marseille, France

**Keywords:** Aerospace engineering, Animal behaviour, Applied physics, Information theory and computation

## Abstract

Attitude control is an essential flight capability. Whereas flying robots commonly rely on accelerometers^1^ for estimating attitude, flying insects lack an unambiguous sense of gravity^2,3^. Despite the established role of several sense organs in attitude stabilization^3–5^, the dependence of flying insects on an internal gravity direction estimate remains unclear. Here we show how attitude can be extracted from optic flow when combined with a motion model that relates attitude to acceleration direction. Although there are conditions such as hover in which the attitude is unobservable, we prove that the ensuing control system is still stable, continuously moving into and out of these conditions. Flying robot experiments confirm that accommodating unobservability in this manner leads to stable, but slightly oscillatory, attitude control. Moreover, experiments with a bio-inspired flapping-wing robot show that residual, high-frequency attitude oscillations from flapping motion improve observability. The presented approach holds a promise for robotics, with accelerometer-less autopilots paving the road for insect-scale autonomous flying robots^6^. Finally, it forms a hypothesis on insect attitude estimation and control, with the potential to provide further insight into known biological phenomena^5,7,8^ and to generate new predictions such as reduced head and body attitude variance at higher flight speeds^9^.

## Abstract (web summary)

Attitude can be extracted from optic flow when combined with a motion model that relates attitude to acceleration direction, which leads to stable flight attitude control with slight oscillations due to unobservable conditions.

## Main

In the fight against gravity, it is crucial for flying robots and animals to control their attitude, thus determining the direction of forces such as thrust and lift. Flying robots can be designed to have a passively stable attitude, meaning that they do not need to actively control their attitude to stay upright. Examples include fixed-wing drones^10^ and tailed flapping-wing robots^11^. However, passive stability comes at a cost, as it requires a minimal velocity and leads to reduced agility. Indeed, agile flyers such as flying insects^12^, quad rotors^13^ and tailless flapping-wing robots^6,14^ are inherently attitude-unstable and rely on active attitude control. To this end, unstable flying robots commonly feature accelerometers^15^, as filtering acceleration measurements over time allows to retrieve the gravity direction^13^.

It is still unclear whether and how flying insects estimate their attitude^3,5,16,17^. Although insects have many different sensory modalities, no specific gravity sensor such as an accelerometer has been found. Sensory cues that carry information on the gravity direction when walking (such as leg loads^18,19^), are not valid when airborne. A flying body is often subject to accelerations larger than gravity in other directions, especially during manoeuvring^20^. Moreover, organs with gyroscopic function such as the halteres in dipterans^3^ can aid stabilization by providing information on body rotation rates, but they carry no information on the absolute attitude angle itself. Depending on the insect species, rotation rates may also be sensed with antennal flagella^21^, wing strains^22^, ocelli^23,24^ or by separating the rotational and translational components of optic flow^25^. In principle, one can integrate rotation rates starting from a known initial attitude^26^, but the estimated attitude will then drift over time.

A few bio-inspired control approaches have forwarded the interesting possibility that insects may bypass estimating attitude altogether^17,24,27^. It has been demonstrated that pendulum-like flapping-wing robots can be stabilized around hover purely by countering rotation rates^24^. A full control system can also use optic flow for controlling flight speed^17,27^. However, the system’s control performance will depend on setting the rotation rates such that the available thrust and lift forces reach the desired directions quickly enough. Because the right sign and magnitude for rate commands depend on the attitude angle, these approaches will also benefit from taking attitude into account.

## Combining optic flow and a motion model

Here, we explore whether the attitude angle can be retrieved when combining optic flow with a motion model. Motion models are commonly used for state estimation in flying robots, but almost always incorporate measurements from an inertial measurement unit, containing gyros, magnetometers and accelerometers, to retrieve attitude^28,29^. A few studies have attempted to estimate attitude angles with just optic flow and motion models before^30–33^. However, the results from these studies are inconclusive. First it was shown that attitude angles could not be determined in this manner for fixed-wing drones^30^. Follow-up studies demonstrated that attitude deviations from the forwards flight equilibrium point are observable^31–33^, but already so when observing the drone’s rotation rates alone. Indeed, the simulation experiments show growing errors on the pitch angle^32^, indicating that the model may be largely relying on integrating rotation rates.

**Fig. 1:**
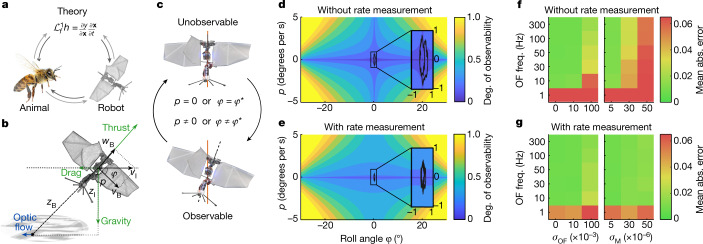
Theoretical analysis proves that attitude can be estimated with optic flow and a thrust-vectoring motion model but that the presence of unobservable states leads to slight attitude oscillations. **a**, Illustration of our approach to studying optic-flow-based flight attitude control. Grey arrows represent the influence of insights and inspiration, and black arrows represent modelling and the generation of hypotheses. ${{\mathscr{L}}}_{f}^{1}h$ is the Lie derivative of the optic flow observation equation. The honeybee image is reprinted with the permission of iStock.com/Antagain.  **b**, Thrust-vectoring motion model of an unstable flying system, that is, robot or insect, and an axis system used for a 2D constant-height model, with body velocities ${v}_{{\rm{B}}},{w}_{{\rm{B}}}$, roll attitude angle $\phi$ and rate $p$, distance along the principal axis, ${Z}_{{\rm{B}}}$, to a world point for which optic flow is measured and inertial velocity ${v}_{{\rm{I}}}$ and altitude ${Z}_{{\rm{I}}}$. **c**, Illustration showing that the proposed approach to attitude estimation leads to a continuous transition between observable and unobservable states, leading to slight attitude oscillations of the system. **d**, The degree (deg.) of observability (equation (35), Supplementary Information) in a part of the state space for a constant-height model without rate measurements, with the remaining variables set to ${v}_{{\rm{I}}}=0$, ${Z}_{{\rm{I}}}=1$ and moment $M=0$. The colour range goes from unobservable (dark blue) to higher degrees of observability (yellow), which implies a faster convergence of a state estimation filter. The state is unobservable if the system is upright ($\phi =0$) or not rotating ($p=0$). A state space trajectory is shown of a controller with as desired state ${\phi }^{* }=0$ (black solid line in the plot’s centre and in the inset). **e**, The same graph for a constant-height system with rate measurements. The state is now only unobservable in the case of zero rate. **f**, Control performance for the constant-height system without rate measurements. The figure shows the mean absolute (abs.) error $\bar{|{\omega }_{y}-{\omega }_{y}^{\ast }|}$ for the simulated system over *N *= 10 runs (from green to red). A mean absolute error $\ge 0.05$ means that the controller is not able to track the reference. The *y* axis represents the optic flow sensing frequency (OF freq.), and the *x* axis represents different noise settings for the optic flow measurement ${\sigma }_{{\rm{OF}}}$ and actuation noise on the generated moment ${\sigma }_{{\rm{M}}}$, separately. **g**, The same graph as **f** but for a constant-height system with rate measurements.

We follow a bio-robotics approach (Fig. 1a) to studying optic-flow-based attitude estimation and control. First, we prove theoretically that attitude angles can be estimated when combining optic flow measurements with a generic, thrust-vectoring motion model of unstable flyers. This type of model relates body attitude, that is, pitch and roll angles, to acceleration direction. It applies to rotorcraft such as quad rotors^13^, but also to insects^34–36^ and tailless flapping-wing robots^6,14^ when averaging forces over the flapping cycle. Mathematically describing the sensory inputs and the motion model enables a formal analysis of the state’s ‘observability’. The state of the two-dimensional (2D) model in Fig. 1b is a vector with the roll angle, velocities and height, whereas its sensory input comes from a single optic flow sensor similar to an elementary motion detector^37^, directed downwards from the body. The state is observable if it can be uniquely determined by tracking motor actions and sensor observations over time.

We investigate the thrust-vectoring model for various levels of complexity, starting from a basic constant-height model without drag (Theoretical analysis and Supplementary Information). Non-linear observability analysis shows that the state, including the attitude angle, is locally, weakly observable^38^. This means that at a single time instant, changes in the observation and corresponding time derivatives can be uniquely linked to changes in the state. A further mathematical and numerical analysis indicates that the model even possesses the stronger property of local observability, indicating that the state itself can be determined instantaneously.

However, the observability depends on the values of the state variables and control inputs. To illustrate this, Fig. 1d,e shows the degree of observability (equation (35), Supplementary Information) for two variants of a constant-height model, in which a higher degree implies that changes in the state can be observed more easily. The model in Fig. 1d estimates rotational accelerations generated by its motor actions, whereas the model in Fig. 1e also measures the rotation rate. The latter model’s degree of observability is higher throughout the state space, but both models have an unobservable state when the roll rate *p* = 0° per s. At first, this seems to represent a considerable problem as a zero rate will occur frequently, that is, whenever the controller reaches its target attitude angle or optic flow setpoint. In engineering, having unobservable states at the core of the control system would be regarded as unacceptable and remedied by adding extra sensors.

By contrast, we propose that nature may have accommodated the unobservability of attitude in certain states. For the basic constant-height model, we provide a proof (Supplementary Information) of the control system’s stability, including the unobservable conditions. It consists of two parts: (1) when the state is observable the controller is able to achieve its control objective, which will lead to zero rate, that is, a condition in which the state is unobservable. (2) When the state is unobservable, noise and disturbances will lead to a condition in which the state is observable again. For example, a direct effect is caused by actuation noise in the moment generation that makes the model rotate, inducing observability. Another example is an indirect effect caused by sensor noise, which will lead to a wrong attitude estimate. Because the wrong estimate will be off-target, the controller will command a ‘corrective’ action that results in a non-zero rate and thus an observable state. Consequently, the system will continuously move into and out of unobservable states, leading to slightly oscillatory motions. This is illustrated in Fig. 1c and the oscillations are evident from the elliptical black line trajectories in $\left(\phi ,p\right)$-space shown on Fig. 1d,e.

Closed-loop simulation experiments with varying noise levels confirm that the unobservable states do not hamper successful attitude or optic flow control. Figure 1f,g shows the control performance for the model without and with rate measurements. In general, the performance benefits from fast vision measurements, as performance increases with an increasing vision update frequency. Moreover, the control performance is worse for the model without rate measurements in which increasing actuation noise forms a problem. These simulation results show that rotation rate measurements are not strictly necessary for attitude estimation and control, but do improve control performance.

The mathematical and numerical analysis of increasingly complex models shows that their state is also locally, weakly observable. The complexities introduced include a varying height model with drag and wind, imperfect thrust prediction, a sloped surface and finally flight in generic three-dimensionally structured environments (Supplementary Information). Attitude is observable with the help of a thrust-vectoring model as it links attitude to accelerations and acceleration changes that are captured by optic flow and its time derivatives. However, the state is always unobservable in a perfect hover condition, that is, when the attitude is constant and optic flow is cancelled out.

## Robotic experiments

**Fig. 2:**
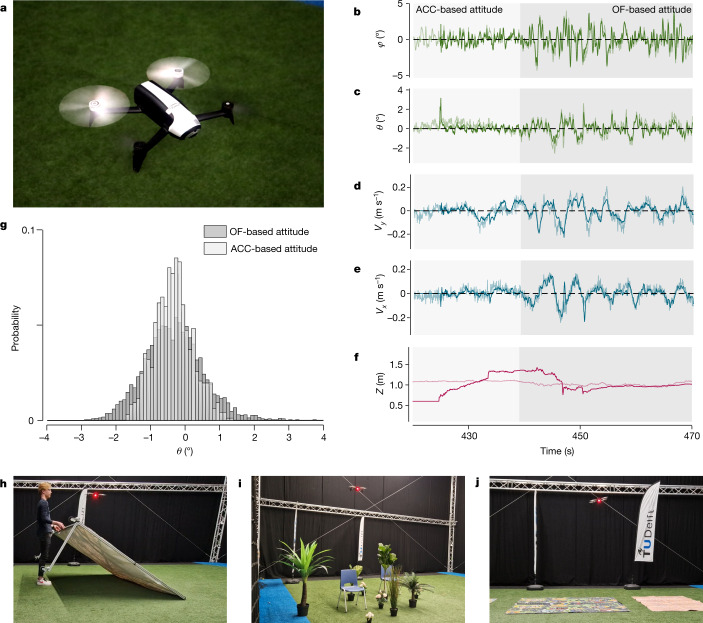
The theoretical findings are confirmed by robotic experiments in which fully autonomous flight is demonstrated based on optic flow and gyro measurements. **a**, Quad rotor robot used in the experiments. **b**, Optic-flow-based (thick line) and accelerometer-based (thin line) estimated roll angles over time during a hover-experiment in which the drone flies first with the accelerometer-based estimate (light grey shading, ‘ACC-based attitude’) and then with the optic-flow-based estimate (dark grey shading, ‘OF-based attitude’). **c**, Optic-flow-based (thick line) and accelerometer-based (thin line) estimated pitch angles over time. **d**, Optic-flow-based (thick line) and motion-tracking-based (thin line) estimated lateral velocity ${v}_{y}$ over time. **e**, Optic-flow-based (thick line) and motion-tracking-based (thin line) estimated longitudinal velocity ${v}_{x}$ over time. **f**, Optic-flow-based (thick line) and motion-tracking-based (thin line) height $Z$ over time. **g**, Comparison of sampled probability distributions of the pitch angle $\theta$ while flying with an accelerometer-based estimate (light grey, foreground) and an optic-flow-based estimate (dark grey, background), data from $N=10$ flights, 5,471 samples. **h**, The drone flying over a moving slope. **i**, The drone flying over a three-dimensionally structured environment. **j**, Disturbance-rejection experiment in which the roll is perturbed by 10°.

**Extended Data Fig. 1:**
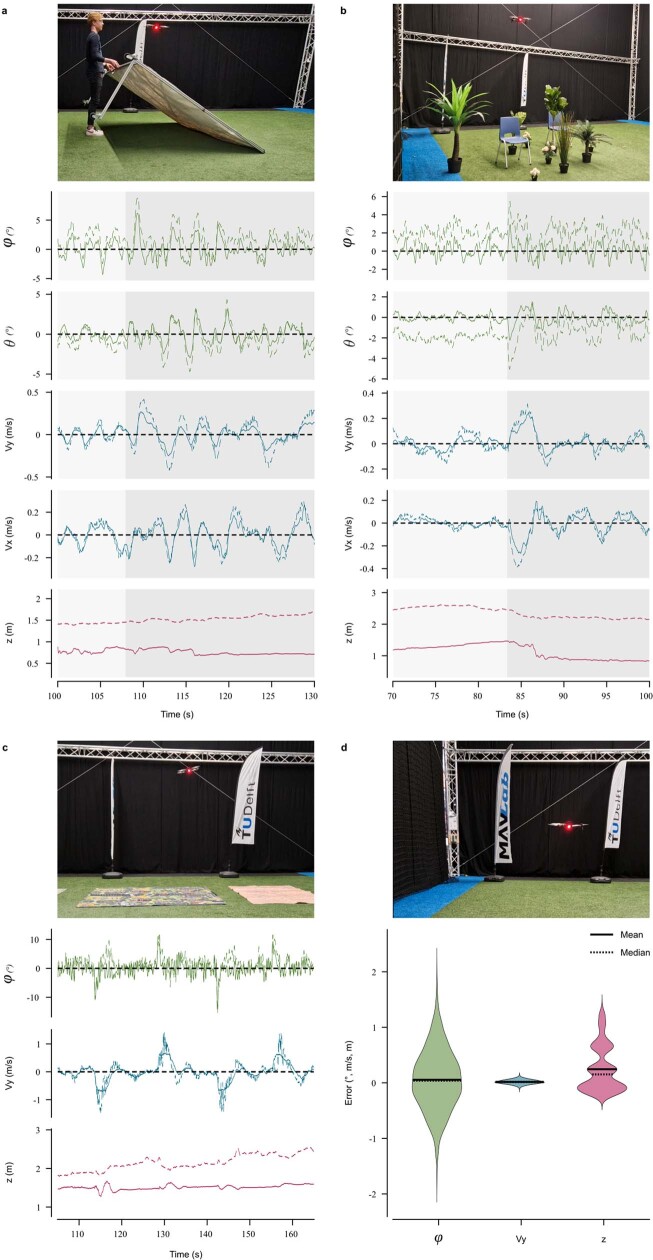
Robustness experiments robotic flight. **a**, Quadrotor flying based on optic flow above a strong slope, which is dragged away, violating the assumption of a static flat ground. From top to bottom, the plots show the estimated attitude angles, velocities, and height. **b**, Quadrotor flying above three-dimensional objects, with artificial plant leaves moving due to the propellers’ downwash, again violating the assumption of a static flat ground. **c**, Quadrotor reacting to a 10-degree roll-disturbance. The plots below show the roll angle, lateral velocity and height over time for four of such disturbances. **d**, Ten subsequent flights are performed to gather statistics on the attitude variation and errors when attitude is estimated with optic flow. The violin plot shows the error statistics over the ten flights for the estimated roll angle, lateral velocity, and height.

Experiments with a free-flying, fully autonomous quad rotor (Fig. 2) confirm the theoretical findings. The drone observes both the longitudinal and lateral ventral optic flow, capturing the ratio of the horizontal velocities and the height, and the optic flow divergence, representing the ratio of the vertical velocity and the height (Quad rotor experiments). Its objective is to hover, eliminating ventral flow by estimating and controlling the roll and pitch attitude angles and divergence by means of thrust control. When flying with a traditional complementary filter based on gyros and accelerometers^1^, the drone hovers still (${\sigma }_{\phi }=0.96$, ${\sigma }_{\theta }=0.55$, Fig. 2g). Switching to the proposed attitude estimation scheme using optic flow and gyros, indeed leads to slight oscillations, as is evident from the attitude angles and velocities over time in Fig. 2b–f and the wider angle histogram in Fig. 2g (${\sigma }_{\phi }=1.24$, ${\sigma }_{\theta }=0.84$, significantly different from accelerometer-based flight with $P < 0.001$, two-sided bootstrap method^39^). Furthermore, the height is most difficult to estimate (Fig. 2f and Extended Data Fig. 1d). We note, however, that neither the estimated velocity nor the height is used by the drone’s control loops. Instead, the drone directly uses optic flow measurements. In general, the attitude estimation and control of the robot is very robust, despite the assumptions of a constant height and flat ground. This is shown by more experiments with slopes or three-dimensional (3D) structures under the drone and with angle disturbances (Fig. 2h–j and Supplementary Videos 1–8). Similar results have been obtained with a varying height model (Supplementary Information and Extended Data Fig. 6). The robustness is partly due to the drone processing optic flow over the entire flow field (Quad rotor experiments).

**Fig. 3:**
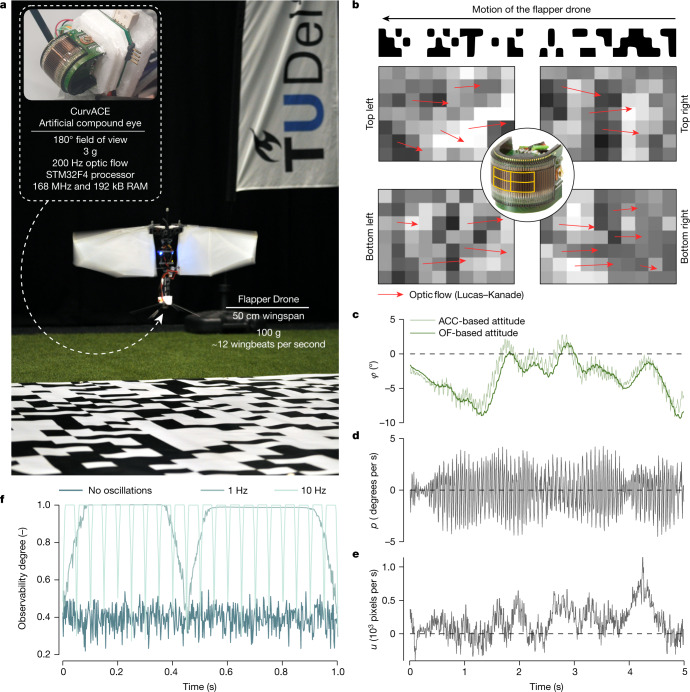
Experiments with a bio-inspired flying robot show that residual oscillations from flapping-wing motion improve observability. **a**, Flapping-wing robot experiment, featuring a 50 cm wingspan ‘flapper drone’ (design based on ref. ^14^) carrying the light-weight, high-frequency artificial compound eye CurvACE^40^. A constant-height model was implemented that only used lateral ventral flow (no divergence). **b**, The CurvACE determined optic flow at 200 Hz in four separate downwards facing regions in its field of view. Each time instance it used one step of the Lucas–Kanade optic flow algorithm ﻿to determine the flow in the *x* and *y* directions at these four locations. During the experiments, the lateral optic flow was determined by averaging the flow in the *x* direction over the four areas. **c**, Estimated roll angles over time during one of the experiments, estimated by a complementary filter that uses the accelerometers (thin line) and by a filter that is based on optic flow and gyro measurements (thick line). There is no ground truth $z$ or ${v}_{y}$, as the motion-tracking system needed to be switched off as its infrared lights influenced the CurvACE sensor. **d**, Roll rate over time. **e**, Average optic flow over time (in pixels per second). **f**, Simulation results for a constant-height model, in which we compare the default case (dark blue) with cases in which we actively add sinusoidal oscillations of different frequencies to the roll rate (1 Hz, medium blue, 10 Hz, light blue line). The observability degree increases substantially due to the higher rotation rates.

To better approximate natural flyers, we also performed experiments with a bio-inspired flapping-wing robot (Flapping-wing robot experiments, Fig. 3a). The robot is equipped with an artificial compound eye called CurvACE^40^ (Fig. 3b). It features a wide field of view of 180° × 60° with a coarse visual resolution of 40 × 15 pixels. We determine optic flow in four regions at a high temporal resolution of 200 Hz, close to the flicker fusion frequency of honeybee vision^41^. We initially thought that the residual flapping-wing motion on the compound eye would hamper state estimation (see the rates and optic flow in Fig. 3d,e). However, the optic-flow-based attitude estimates correspond well to those of the complementary filter using accelerometers (Fig. 3c). We subsequently realized that the residual flapping motion did not impair but improved attitude observability. Figure 3f shows that oscillations are beneficial to observability, with higher frequencies shortening the time duration of low observability. This finding suggests that flying insects or robots could benefit from residual flapping-wing oscillations or even actively induce rotation rates to enhance the degree of observability—in the spirit of active vision^42,43^.

## Discussion

Our findings have implications for robotics. First, tiny, insect-sized flying robots such as the Robobee^6,44^ are extremely resource-limited. For such robots, even small MEMs-based sensors form a burden. We have demonstrated that accelerometers are not necessary to successfully control attitude. Second, most autopilots for flying robots only incorporate lateral ventral flow into their state estimation. We have shown that optic flow divergence can improve redundancy, even allowing to fly completely autonomously without any height sensors or accelerometers. Third, accommodating unobservability is a strategy with broader implications than optic flow control alone. For instance, wireless-ranging-based relative localization in drone swarms^45^ leads to important unobservable conditions such as during formation flight. The current study suggests investigating the option of a minimalistic system accommodating this unobservability instead of a heavier, more power-hungry system with more sensors.

The presented approach also forms a hypothesis on insect attitude estimation, potentially explaining various phenomena observed in flying insects. First, it explains which role optic flow may play in attitude estimation and control. Optic flow was shown to be essential to hoverflies for stabilizing their flight when falling^5,16^. The hoverflies’ behaviour was best explained by a model that incorporated attitude angles^16^, but it was unclear how such angles were estimated without a clear visual horizon in the environment. We have shown that this is possible if the insect possesses a motion model, relating attitude to acceleration direction. This raises the question of how plausible it is for insects to have a motion model, with which we intend any means to use predicted effects of actions for perception and control. In ref. ^46^ it is argued that insects possess such ‘forwards models’ and that they serve goals such as reducing action latency^47^ and differentiating between external disturbances and expected feedback^48^. Our study highlights another potential purpose of forwards models, that is, to make states such as attitude observable. The implementation of such a model in the brain can be implicit, for example, reminiscent of how visual receptive fields of lobula plate tangential cells seem to be tuned to an insect’s motion model^49^. Second, the results reported in Fig. 2 may explain the (im)precision of flight for different species and conditions. For instance, honeybees can still fly, but less precisely, when their ocelli are covered with opaque paint^8^. Moreover, the results in Fig. 3 indicate a potential usefulness for flapping-induced, high-frequency thorax and head oscillations of blowflies^7^.

**Extended Data Fig. 2:**
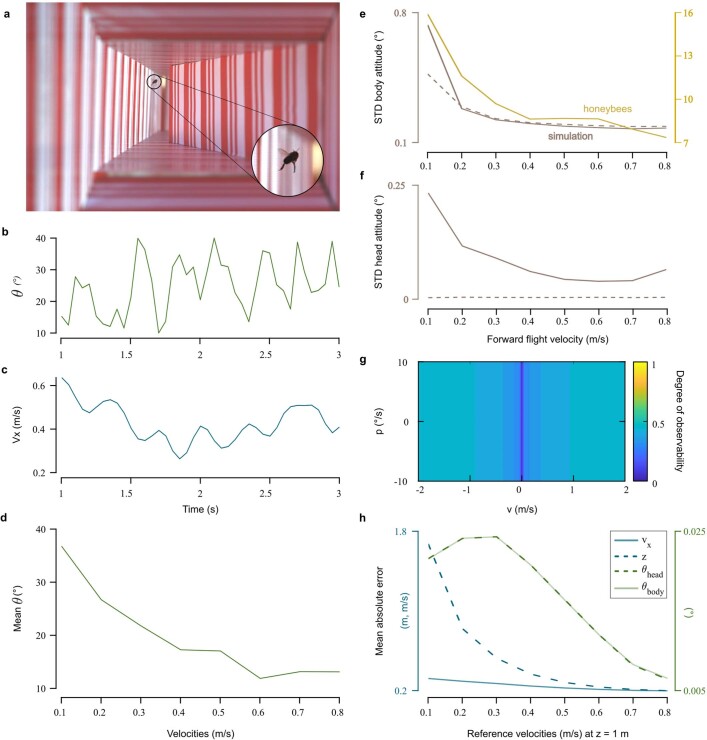
Preliminary analysis investigating the hypothesis that attitude variation decreases when flight speed increases. **a**, Picture of a honeybee flying in the doubly tapered corridor in the experiments of^9^, photo reprinted with permission of DGA / François Vrignaud. **b**, Pitch angle for a single honeybee trajectory over time for one of the trajectories. **c**, Corresponding velocity over time. **d**, Mean pitch angle for the honeybees vs. forward flight speed. **e**, Standard deviations of the body attitude angles per velocity bin, ranging from [0.05, 0.15) m/s to [0.75, 0.85) m/s for 21 honeybee trajectories (dark yellow) and 30 simulation trajectories (light brown). Two different simulation models are compared, one with the proposed optic-flow-based state estimator (solid line) and an alternative model that perceives a noisy version of the ground-truth attitude (dashed line). **f**, Standard deviation of the simulated head attitude angle for different velocities, again for the optic-flow-based state estimator (solid line) and an alternative model that perceives a noisy version of the attitude (dashed line, almost zero). **g**, Degree of observability for a constant height system with drag and both lateral flow ${\omega }_{y}$ and divergence ${\omega }_{z}$ as observations for different velocities and rotation rates. **h**, Mean absolute estimation errors in velocity, height, and body and head attitude angles for different flight velocities in the simulation.

Verifying the hypothesis may be challenging, as it concerns brain processes that are hard to monitor during flight. One potential avenue is to exploit the prediction that the degree of observability changes over the state space, which in turn will affect the insect’s attitude variation. For example, closed-loop simulation experiments with a head-and-body model (Supplementary Information) show that observability increases and attitude variation in both body and head decreases for higher flight speeds. As a preliminary analysis we investigated the biological data from honeybee experiments by Portelli et al.^9^. The data only allow us to retrieve the body pitch angle, which indeed has a lower variance for higher speeds (Supplementary Information and Extended Data Figs. 2 and  9). However, other phenomena also influence this trend. For example, parasitic drag will be larger at higher flight speeds, stabilizing attitude. In the same time, aerodynamic insect models^34–36^ also predict increasing pitch instability at higher flight speeds, destabilizing attitude. More simulation experiments, piecing apart parasitic drag from observability effects, suggest that only observability affects the trend of the head attitude (Supplementary Information). Future biological studies that track not only body but also head attitude or that manipulate sensory inputs could give further insight into this matter.

Finally, one can wonder what role the proposed mechanism plays in the context of insects’ many more sensory cues. On the one hand, adding more sensors will improve the observability. On the other hand, unless such further sensory cues directly encode for the gravity direction, flight conditions such as a pure hover will remain unobservable. Hence, the main findings on unobservability and the ensuing attitude variations stay relevant when taking into account extra senses. Because animals generally rely on redundant information sources, even larger animals such as birds could use optic flow and motion model information to support their attitude estimation^50^.

## Methods

### Theoretical analysis

The theoretical analysis of the observability of the state, including attitude, relies on both a motion model and a model of the sensory inputs. In this section, we first explain the model for the elementary case of a quad rotor flying at a constant height above a flat ground surface. The model captures the main characteristics necessary for attitude estimation with optic flow, while leading to mathematical formulas of limited complexity and hence improved comprehensibility. Subsequently, we discuss more general models of motion and more generic environments. The mathematical derivations and formulas involved in the non-linear observability analysis and stability proof for the constant-height model are detailed in the Supplementary Information for brevity.

### Constant-height model

#### Observability analysis

**Extended Data Fig. 3:**
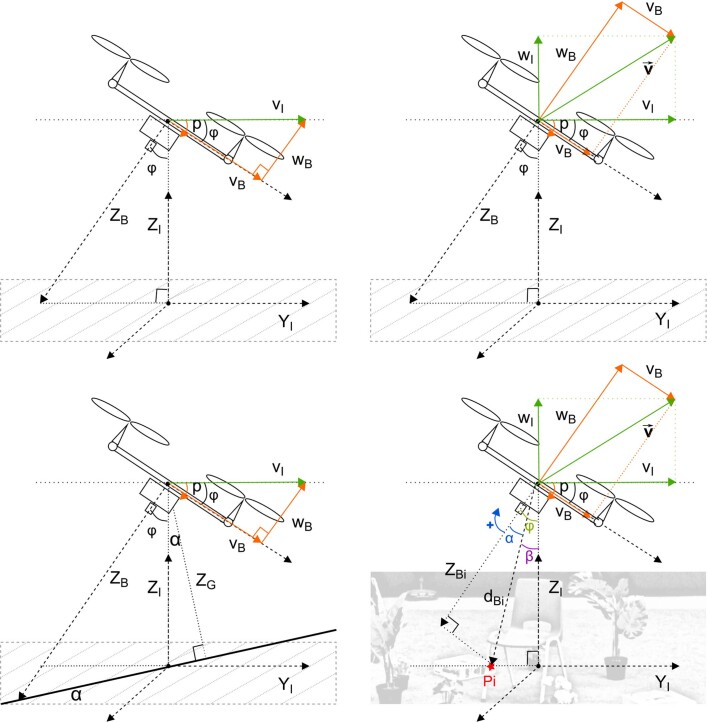
Sketches of different quad rotor and environment models. **a**, Axis definitions for a constant height quadrotor model. ${\rm{B}}$ indicates the body frame, whereas $I$ indicates the inertial frame. The arrows for $Y$ and $Z$ point into the positive directions. The attitude angle $\phi$ represents the quadrotor’s roll angle, and $p$ the roll rate. The shaded rectangle represents the floor. **b**, Varying height model of a quad rotor in the 2D plane. **c**, Constant height motion model of a quad rotor where the ground has slope angle $\alpha$. **d**, Varying height model in the 2D plane for a drone flying over an uneven terrain. The drone uses one world point ${P}_{i}$ (red star) for state estimation.

Without loss of generalization with respect to a thrust-vectoring model, we will consider a quad rotor drone’s motion in the 2D plane. Please see the axis definitions in Extended Data Fig. 3a. In our analysis, we focus on the roll angle $\phi$ (and roll rate $p$), but the findings are equally valid for the pitch angle $\theta$ (and pitch rate $q$). In practice, estimating pitch instead of roll may require different parameters for drag and moment of inertia in the case of an asymmetric body. As a result, the stability properties of these axes may be different, but this does not fundamentally affect the analysis. The velocity in the inertial *z* axis will be denoted with ${w}_{{\rm{I}}}$ and that in the inertial *y* axis with ${v}_{{\rm{I}}}$. In Extended Data Fig. 3a, ${w}_{{\rm{I}}}$ is not shown as it is zero. For velocities in body axes, we will use ${w}_{{\rm{B}}},{v}_{{\rm{B}}}$, for the body *z* and *y* axes, respectively.

The observation model represents the optic flow in the direction of the camera’s principal axis. For our derivations, we use a pinhole camera model. We are interested in the time derivative of the feature’s location in the camera’s field of view, which at the principal axis image coordinate, $(x,y)=(\mathrm{0,0})$, is given by^51^:

1 $${\omega }_{y}=-\,\frac{{v}_{{\rm{B}}}}{{Z}_{{\rm{B}}}}+p=-\,\frac{{\cos }^{2}\left(\phi \right){v}_{{\rm{I}}}}{{Z}_{{\rm{I}}}}+p$$

where ${\omega }_{y}$ is the ventral lateral flow. Equation (1) is valid for the interval $\phi \in \left(-9{0}^{^\circ },9{0}^{^\circ }\right)$, where the parentheses denote the exclusion of the interval borders. The right-hand side of equation (1) is based on geometric relations visible in Extended Data Fig. 3a that would change if the roll angle were outside this interval.

The state is defined as a vector ${\bf{x}}=[{v}_{{\rm{I}}},\phi ,{Z}_{{\rm{I}}}]$, and the control input (motor action) is the roll rate, that is, $u=p$. This leads to the state update equation, with *g* representing the gravitational acceleration:

2 $$f({\bf{x}},u)=\left[\begin{array}{c}{\dot{v}}_{{\rm{I}}}\\ \dot{\phi }\\ {\dot{Z}}_{{\rm{I}}}\end{array}\right]=\left[\begin{array}{c}g\,\tan \left(\phi \right)\\ p\\ 0\end{array}\right]$$

Equations (1) and (2) form the basis for the non-linear observability analysis, of which the details can be found in the Supplementary Information. The analysis shows that the system is locally, weakly observable in most of the state space. Weak observability implies that given the sensory input and its time derivatives, changes in the state can be uniquely identified. Local stands for local in time, that is, the estimation can be done at a single time instant. The main condition in which the state is unobservable (not weakly, locally observable), is when the roll rate is zero, $p=0$. This condition corresponds to flying with a constant roll angle, in which the acceleration is not changing, that is, there is no ‘jerk’. We also analyse the stronger property of local observability for this model. The theoretical and numerical analysis indicate that in most of the state space the system is locally observable, that is, that the sensory input and its time derivatives suffice for directly determining the state. The two main conditions for which the state is locally unobservable are $p=0$ and $\phi =0$, that is, when there is either no jerk or no acceleration.

#### Control system stability

At first sight, the unobservable condition of $p=0$ may seem problematic because an attitude controller that reaches the desired attitude will set the rate to zero. Hence, if the control system is successful, it will lead to unobservability of the system. In the Supplementary Information, we provide a stability proof for the constant-height model, which takes conditions into account in which the state is unobservable. The first part of the proof shows that when the state is observable, the control will be able to reach a desired attitude angle ${\phi }^{* }$. If this angle is reached, the controller will command $p=0$, which leads to unobservability of the system. The second part of the proof shows that sensor noise, actuation noise or external disturbances will always make the system observable again.

#### Simulation setup

The proof is supported by evidence from simulation experiments (Supplementary Information and Extended Data Figs. 7 and 8). Here we explain the simulation setup, as simulations with different models also follow the same scheme (for example, the simulation results in Fig. 1 and Extended Data Fig. 2). The simulation uses the motion model in equation (2) for the evolution of the ground-truth state over time. It also features a simulated ‘robot’ that receives optic flow observations according to equation (1), but delayed and with additive Gaussian noise: ${\hat{\omega }}_{y}\left(t+\bigtriangleup t\right)=-\,\frac{{\cos }^{2}\left(\phi (t)\right){v}_{{\rm{I}}}(t)}{{Z}_{{\rm{I}}}(t)}+p(t)+\mu (t+\bigtriangleup t)$, with $\triangle t$ as the delay and $\mu  \sim {\mathscr{N}}\left(0,{\sigma }_{{\omega }_{y}}\right)$ the noise, where the tilde (~) means "distributed as". These observations are input into an extended Kalman filter (EKF)^52^, which uses equation (2) for predictions and linearization of equation (1) around the current estimated state $\hat{{\bf{x}}}$ as the observation equation. The simulated robot has a proportional, integral ‘outer loop’ controller for reaching a desired optic flow value ${\omega }_{y}^{* }$. The output of this controller is a desired roll angle, ${\phi }^{* }$. An ‘inner loop’ proportional integral controller then sets the rate command $p(t)$ on the basis of the error in the roll angle, that is, the difference between the desired and estimated roll angle $({\phi }^{* }-\hat{\phi })$. Whereas the EKF uses this commanded roll rate for its predictions, it is used in the simulator after being delayed and perturbed by Gaussian noise. Thus, the $p$ entered in equation (2) is $p(t+\bigtriangleup t)=p(t)+\mu (t+\bigtriangleup t)$, with $\mu  \sim {\mathscr{N}}\left(0,{\sigma }_{p}\right)$.

#### Model extensions

The two central assumptions of the elementary constant-height model may sound stronger than they actually are. First, as we perform local observability analyses, the flat ground assumption only needs to hold close to the world point now perceived by the optic flow sensor (spatially local flatness). Moreover, although the height is assumed constant, it is part of the state that is estimated. Hence, height changes will eventually be picked up by the state estimation. Nonetheless, we also study extensions of the model both in terms of motion and structure of the environment. Below we briefly discuss the various extensions, of which the details can be found in the Supplementary Information.

First, in the analysis above, *p* is a control input that is known to the system. However, real-world systems such as drones and flying insects do not control attitude rate directly. Instead, by varying rotor speeds or wing flapping amplitudes, they generate moments. Modelling the system as such makes the rate $p$ a state that is to be estimated. The rotation rate can be measured by means of gyros, which gives a very high update frequency (typically $\gg$ 500 Hz), as is done in our robotic experiments (Flapping-wing robot experiments and Quad rotor experiments). It can also be measured with other sensors. For example, it can be extracted from the optic flow field^51^. The disadvantage of this is that the rates are then determined at a lower update frequency, leading to slower, less accurate state estimates. Still, theoretically, measuring *p* is not necessary because predicting the moments caused by control inputs suffices, as shown in the Supplementary Information. This is the motion model that was used for the simulation results from Fig. 1 in the main article. These simulation experiments follow the same simulation scheme as explained for the rate-based constant-height model explained above, except for the state update equations and control being different. Specifically, in these simulations the motor actions of the simulated robot do not consist of rotational rates, but of moments. This leads to the following state update equation: $f({\bf{x}},u)=[{\dot{v}}_{{\rm{I}}},\dot{\phi },\,\dot{p},{\dot{Z}}_{{\rm{I}}}]=[\,g\tan (\phi ),p,M\,/\,I,0]$, where $M$ is the moment and $I$ is the moment of inertia. In this case, the control input (motor action) is the moment, $u=M$, which is also delayed and perturbed by Gaussian noise when performing simulations.

Second, the constant-height model has an obvious potential flaw: can the system keep the height constant enough when it has to be estimated? In practice, this model works well because keeping a roughly constant height is possible through appropriate optic flow divergence control. Still, in the Supplementary Information, we extend the model above to a varying height model (including vertical body velocity), with drag and wind (see Extended Data Fig. 3b for a graphical illustration of the model). Non-linear observability analysis shows that the state of this varying height model, including the current wind velocity, is locally, weakly observable. The state becomes unobservable when we set the thrust to compensate for gravity, the velocities to match the wind and the moment and rate to zero. This setting corresponds to a condition of a pure hover in this model—without accelerations of jerk.

Although this extensive model is still locally, weakly observable, state estimation performance will benefit from further measurements. That is why we also study a varying height model including an extra sensory input, that is, the optic flow divergence, which captures the vertical body velocity relative to the distance to the ground $\frac{{w}_{{\rm{B}}}}{{z}_{{\rm{B}}}}$. This model, which includes drag and a thrust bias as state variables but excludes wind, is described and studied in the Supplementary Information. It is again locally, weakly observable and has been successfully implemented onboard of a quad rotor for robotic experiments (Quad rotor experiments and Extended Data Fig. 6).

Third, we analyse cases in which the ground is not flat. In the Supplementary Information, we investigate what happens when the ground surface is sloped, while still only observing optic flow at the principal axis coordinate (Extended Data Fig. 3c). The state, including the slope angle, turns out to be locally, weakly observable even with this elementary optic flow measurement. Subsequently, in the Supplementary Information we analyse the case of a generic environment with the system having access to the entire optic flow field (Extended Data Fig. 3d). It is well-known that from the entire optic flow field the system can estimate a unit-vector for velocity ${\bf{v}}$, with $\parallel {\bf{v}}\parallel =1$, the rotation rate $p$ and all inverse depths $\frac{1}{{z}_{{\rm{B}}i}}$ for all world points ${P}_{i}$ in view^53^. Finally, in the Supplementary Information it is shown that this suffices for retrieving attitude, velocity and height with respect to a selected point ${P}_{i}$.

Fourth, in all above cases, the eye is rigidly fixed to the body, whereas insects can move their head with respect to their body to stabilize their gaze. In the Supplementary Information we study a head-and-body model, in which the body attitude influences the thrust direction and the head attitude the looking direction (Extended Data Fig. 10). Also this more complex model is locally, weakly observable. This model is used in simulation for the comparison with the biological data (Extended Data Fig. 2).

### Quad rotor experiments

**Extended Data Fig. 4:**
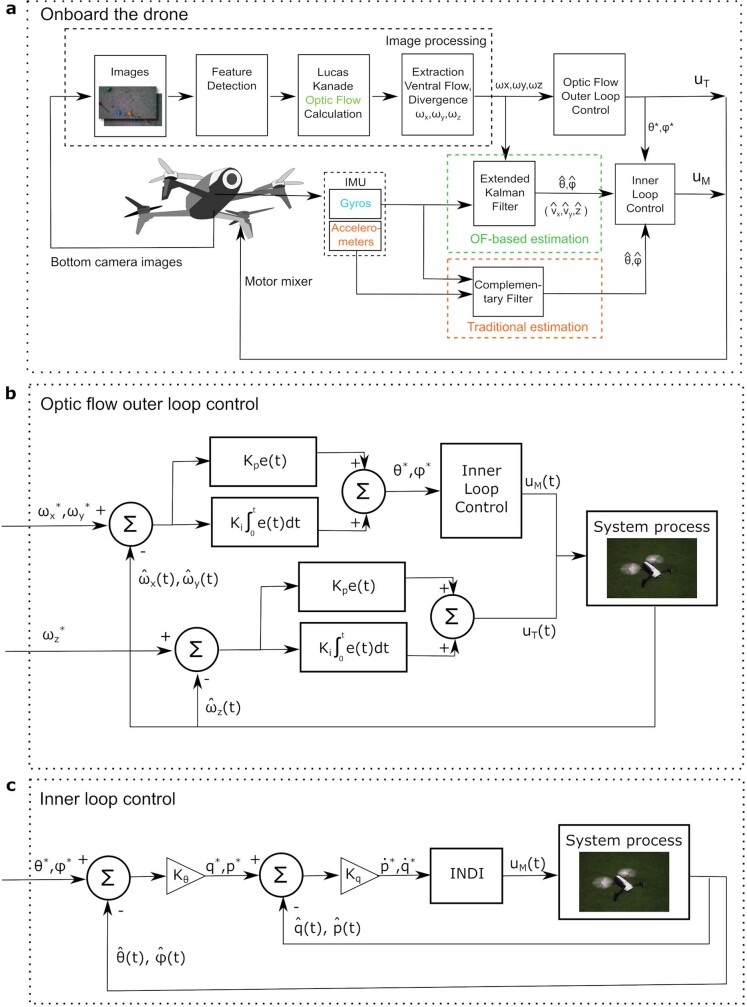
Processing and control loops onboard of the quadrotor drone (a Parrot Bebop 2) used in the robotic experiments. **a**, Onboard image processing, state estimation, and control used for fully autonomous flight of the drone. **b**, Control diagram for the optic flow outer loop control, where the longitudinal and lateral optic flow ${\omega }_{x}$ and ${\omega }_{y}$ are used to set desired attitude angles for the inner loop control and the divergence ${\omega }_{z}$ directly leads to a thrust command ${u}_{T}$. **c**, Inner loop control to transform desired attitude setpoints ${\theta }^{* }$, ${\phi }^{* }$ into moment commands ${u}_{M}$ for the quadrotor motor mixing. For the INDI block we refer the reader to^59^.

The setup for the quad rotor experiments is shown in Extended Data Fig. 4. We use a Parrot Bebop 2 drone for the experiments, replacing its firmware autopilot with the open-source Paparazzi UAV software^54^. All sensory processing and control runs onboard the drone. Here we discuss all processes shown in the figure.

#### Image processing

The image processing pipeline consists of: (1) feature detection with ACT-corner^55^, (2) optic flow determination with the Lucas–Kanade algorithm^56^ and (3) extraction of optic flow measurements $({\omega }_{x},{\omega }_{y},{\omega }_{z})$. The first two represent the longitudinal ventral flow ${\omega }_{x}=\frac{{u}_{{\rm{B}}}}{{z}_{{\rm{B}}}}$ and lateral ventral flow ${\omega }_{y}=\frac{{v}_{{\rm{B}}}}{{z}_{{\rm{B}}}}$. The last one is the optic flow divergence ${\omega }_{z}=\frac{{w}_{{\rm{B}}}}{{z}_{{\rm{B}}}}$. These measurements are obtained from the optic flow field with the methods from ref. ^57^, in which the optic flow is not derotated. The optic flow processing makes a robust fit of the flow field, assuming that it is predominantly linear. Moreover, the calculation of divergence ${\omega }_{z}$ is based on a separate process that estimates size changes in the image, making it insensitive to rotation rates.

#### Optic flow outer loop control

The drone has an optic flow outer loop control, which uses separate proportional integral controllers for the vertical and horizontal axes, as shown with a control diagram in Extended Data Fig. 4b. The vertical axis uses a proportional integral controller for the thrust based on the optic flow divergence error $({{\omega }_{z}^{* }-\omega }_{z})$, in which during our experiments ${\omega }_{z}^{* }=0$, that is, we want the drone to hover. Successful optic flow divergence control requires an appropriate control gain, which in turn depends on the height^57^. Too high a gain will lead to vertical oscillations, which can be detected by the drone and in turn be used to find the right control gain^57,58^. The control gains for lateral control with ${\omega }_{x},{\omega }_{y}$ also depend on height, and we scale them linearly with respect to the vertical control gain. The outer loop lateral and longitudinal control sets the desired attitude angles ${\phi }^{* },{\theta }^{* }$, which are executed by the inner loop attitude controller.

#### Inner loop attitude control

Inner loop attitude control is performed with incremental non-linear dynamic inversion (INDI)^59^. This inner loop controller, illustrated in Extended Data Fig. 4c, uses the errors between the estimated and desired states $({\phi }^{* }-\hat{\phi }),({\theta }^{* }-\hat{\theta })$. It subsequently uses proportional gains to set desired attitude rates and then rotational accelerations. The INDI block that determines the correct moment commands ${u}_{{\rm{M}}}$ to the motor mixing, relies on rotational accelerations that are calculated by low-passing and differentiating gyro measurements. For the exact details of INDI we refer the reader to ref. ^59^.

#### EKF/complementary filter

The attitude estimates used by the inner loop control can either come from an EKF that uses the proposed approach and combines optic flow with gyro measurements, or from a traditional complementary filter that fuses accelerometer and gyro measurements. We can switch between these estimators for use by the control, but always log both estimates for comparison purposes. The EKF is instantiated by using the state and observation equations in our models.

The EKF has parameters for the observation and actuation noise, forming the diagonal entries in the matrices $R$ and $Q$. Moreover, the varying height model includes four parameters that map the four commanded rotor speeds linearly to the thrust value, that is, *T* = ${{\bf{p}}}^{\top }{\bf{r}}$, where **p** is a vector with the four parameters and **r** a vector with the commanded rotor speeds. Although these EKF parameters can be estimated in a supervised manner from data, we obtained the best results by using an evolutionary optimization algorithm, covariance matrix adaptation evolutionary strategy (CMA-ES)^60^. Specifically, we performed seven flights in which we made a high-frequency log of all onboard sensor data. This allowed to run the EKF offline on the data sets. Then, CMA-ES optimized the parameters of the EKF, with as cost function the sum of squared errors of the estimates (comparing EKF estimates with the logged ‘ground truth’ from the complementary filter for attitude and motion-tracking system for height and velocities). Once optimized, the parameters resulted in successful state estimation and did not have to be adapted anymore for any of the test flights presented in the article’s results.

The experiments presented in the main article and Fig. 2 are based on the constant-height model with rotation rate inputs presented in Theoretical analysis. Instead of predicting the rotation rates, gyro measurements are used as a stand-in for the control input to the filter. Moreover, the real robot always also uses the optic flow divergence as an observation. The same model is used for roll and pitch, assuming decoupled dynamics. We also performed experiments with a ‘varying height model’, which only estimates the roll angle but does take into account height changes, as explained in the Supplementary Information (results in Extended Data Fig. 6). Finally, we use the ‘quaternion complementary filter’ implemented in the open-source Paparazzi autopilot^54^ as the standard, accelerometer-based attitude estimation algorithm.

#### Experimental setup: slope

There are several ways in which the robot could take into account a sloped surface, for example, by means of an improved vision or state estimation process (Supplementary Information). However, we also perform an experiment in which we test on the drone what happens if the slope is not taken explicitly into account. Specifically, the drone uses the constant-height model for roll and pitch (Theoretical analysis), which does not include the slope in the state, and the vision processes described above, where the determination of ventral flow and divergence also do not take slope into account. The experimental setup and resulting state estimates are shown in Extended Data Fig. 1a. The screen starts out at a tilt of roughly 20°, but during the experiment it is moved slowly up to an angle of roughly 40° (Extended Data Fig. 1a) and then down again. It turns out that the presence of a slope is not particularly problematic for state estimation, even if it is ignored by the vision processing and in the state estimation setup. When moving up-slope (left in the picture), the optic flow should increase quicker than expected and the angle should be estimated larger. When moving down-slope, the optic flow increases slower than expected, which should lead to a smaller angle estimate. In the case of commanded hover flight, these effects only lead to slightly increased attitude variation (${\sigma }_{\phi }=2.0^\circ$, ${\sigma }_{\theta }=1.54^\circ$), with the estimates still closely resembling the accelerometer-based estimates (Extended Data Fig. 1a). Moreover, during the experiment, the screen that forms the slope is dragged away, which represents a disturbance that is successfully handled by the drone; as it is commanded to keep the lateral ventral flow zero, it moves along with the object. The experiment is included in the Supplementary Videos 1–8.

#### 3D structure

In the Supplementary Information, we show that the proposed approach to attitude estimation does not rely on the ground being a flat surface. We explain there that one can deal with irregular environment structure by using a general vision method to separate the environment’s 3D structure from the ego-motion. However, we also perform an experiment to test whether the constant-height model and the current vision processing are sufficiently robust to deal with a certain amount of 3D structure, by having the drone fly above several objects. The setup for this experiment and corresponding results are shown in Extended Data Fig. 1b. The roll and velocity estimates correspond well to the ground truth. The height seems underestimated, which here could be partly because the objects in view are actually closer to the drone than the ground. During the experiment, the drone first hovers above these objects and then also gets non-zero outer loop optic flow commands (${\omega }_{y}^{* }$) to translate left and right over the 3D structure (as can be seen in the Supplementary Videos 1–8). The attitude is well estimated throughout the experiment. We expect that the robustness of the current method stems from the fact that flow from the entire field of view is integrated to determine the optic flow observation.

#### Disturbance

A disturbance experiment was performed to test the response of both the state estimation filter and optic flow control. Specifically, to create a disturbance, we add a given number of degrees to the desired roll attitude ${\phi }^{* }$ that is determined by the outer loop control. For clarity, the outer loop control is unaware of this addition. As a consequence of this disturbance, which is 10° in our experiments, the inner loop control will command a much larger angle than desired by the outer loop control. The drone will accelerate sideways, leading to a larger lateral ventral optic flow. The outer loop proportional integral controller will attempt to eliminate the flow, with the integral term eventually cancelling out the introduced addition.

#### Several flights

The main paper shows results from ten subsequent flights (Fig. 2g). For each flight, the drone takes off, hovers according to its accelerometer-based attitude estimate, switches to using the optic-flow-based attitude estimate and then lands again. Extended Data Fig. 1d shows a picture of the experimental setup. Please note that during the experiments the ground surface of the arena was not changed to add visual texture. Furthermore, Extended Data Fig. 1d contains the error distributions for the different estimated states during all ten flights, when the drone was using the estimated angles for control. Here, the roll angle is compared to the accelerometer-based roll estimate, which we consider as ground truth. The velocity and height are compared to measurements by the motion-tracking system. It can be seen that both the roll angle and velocity are estimated accurately. The height error distribution is ‘strange’, showing that it is the most difficult variable to estimate, and that around hover the height does not always converge to the correct value. Also, other experiments have shown the height estimates to be the least accurate.

### Flapping-wing robot experiments

**Extended Data Fig. 5:**
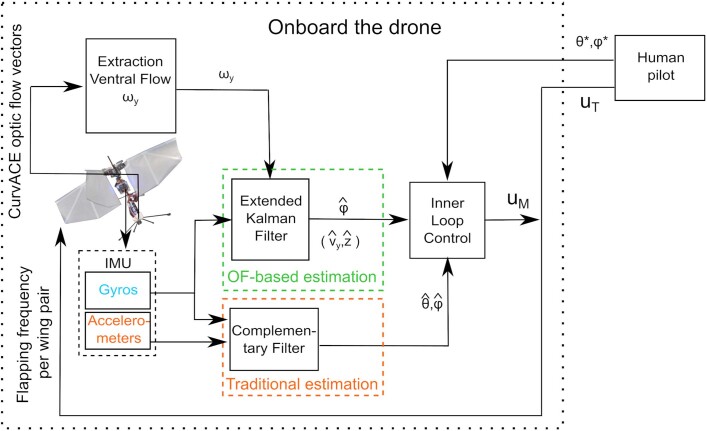
Setup of the robotic experiments with the flapping wing drone (a modified Flapper Drone). A human pilot executes the outer loop control, while the drone itself runs the inner loop attitude controller, based on the onboard roll angle estimates. See the text for details.

**Extended Data Fig. 6:**
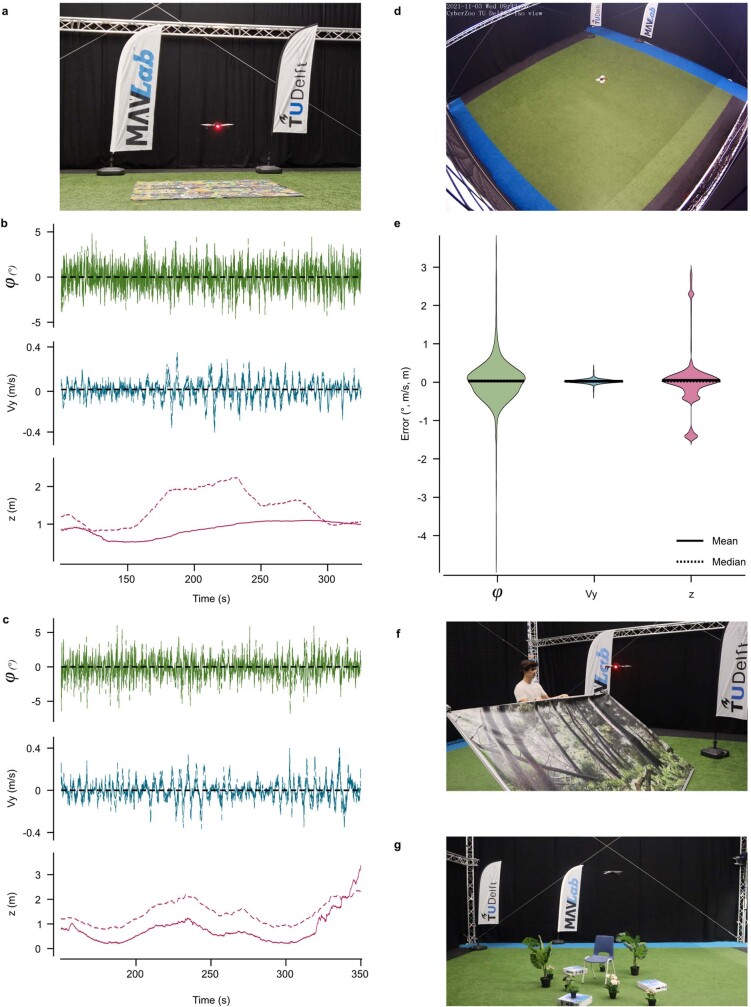
Robotic experiments with height changes. **a**, Picture of the experiment. **b**, Roll angle, lateral velocity, and height over time during the experiment, in which the height was varied, all with the constant height model. Solid lines represent estimates, dashed lines the ground truth. **c**, Roll angle, lateral velocity, and height over time during the experiment, in which the robot estimates and controls its roll axis with the help of the varying height model explained in Supplementary Information. Solid lines represent estimates, dashed lines the ground truth. **d**, Picture of experimental setup in which the drone performed thirteen subsequent flights for gathering state estimation statistics. **e**, Estimation error distributions over the thirteen flights for the roll angle, lateral velocity, and height. **f**, Picture of the quadrotor flying over a slope with the varying height model. **g**, Picture of the quadrotor flying over a 3D structured scene with the varying height model.

**Extended Data Fig. 7:**
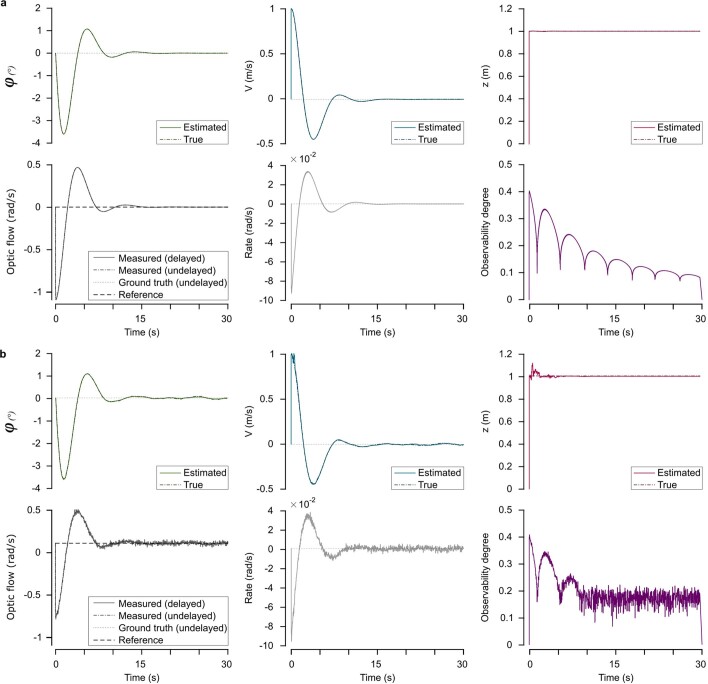
Simulation experiments to verify the stability proof - I. **a**, Simulation without noise. Top row: The three states (true, solid line, and estimated, dashed-dotted line) over time. The dotted line indicates zero. Bottom row, from left to right: The optic flow ${\omega }_{y}$ (solid line) and the reference desired flow (dashed line), the rate (solid line) with a dotted line at zero, and the observability degree over time. **b**, Simulation with sensor noise. Top row: The three states (true, solid line, and estimated, dashed-dotted line) over time. The dotted line indicates zero. Bottom row, from left to right: The optic flow ${\omega }_{y}$ (solid line) and the reference desired flow (dashed line), the rate (solid line) with a dotted line at zero, and the observability degree over time.

**Extended Data Fig. 8:**
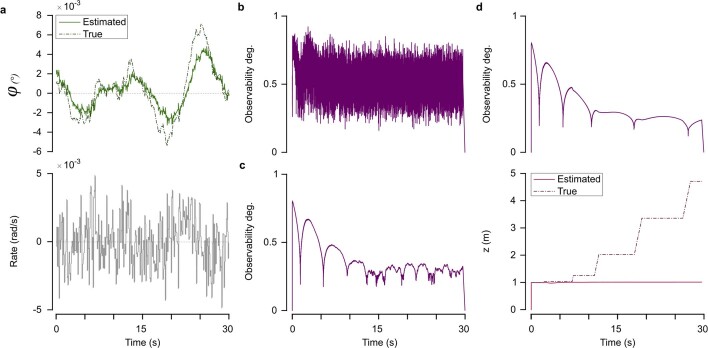
Simulation experiments to verify the stability proof - II. **a**, Zoomed in view on time interval [15,25] seconds for a simulation with sensor noise. Top: True (dashed) and estimated (solid) roll angle over time. Bottom: roll rate. **b**, Observability degree over time for an experiment with actuation noise. **c**, Observability degree over time for an experiment with lateral disturbances. **d**, Experiment with malicious disturbances in the vertical axis when the system is not moving. Top: Observability degree over time. Bottom: True (dashed) and estimated (solid) height over time.

**Extended Data Fig. 9:**
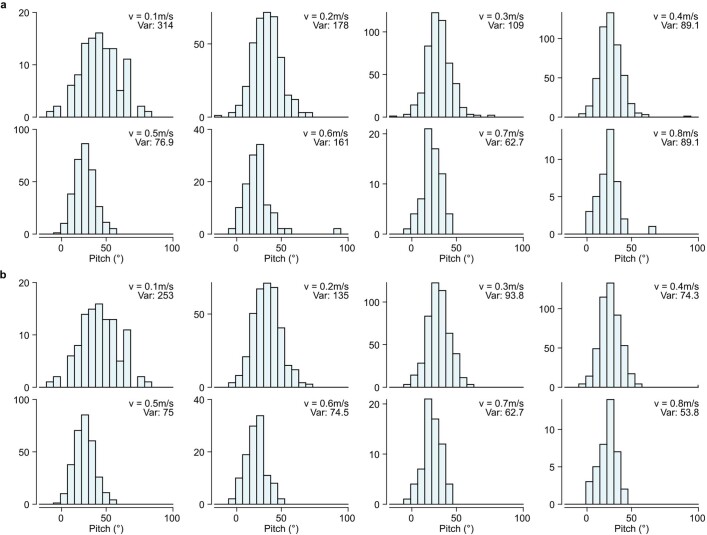
Histograms of honeybee pitch angles at different velocities. **a**, The histograms of all data, i.e., with outliers, of the honeybee body pitch angles for different velocity bins. Each subplot’s title mentions the centre of the speed bin and the variance of the pitch angles in that bin. **b**, The histograms of the data without outliers.

**Extended Data Fig. 10:**
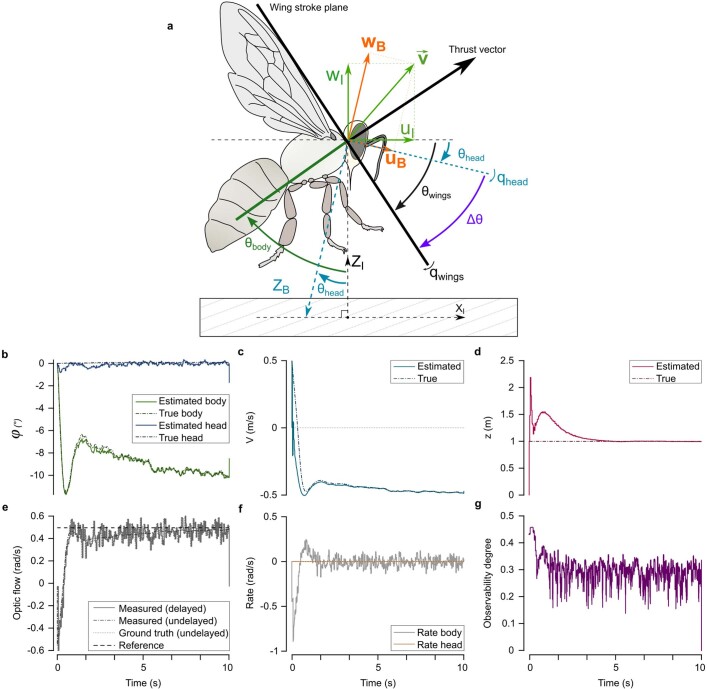
Model with independently moving head and body. **a**, Graphical illustration of a “honeybee” simulation model, in which the head and body rotate independently. The head determines the looking direction of the optic flow sensor, the body determines the thrust direction. For a detailed explanation, see Supplementary Information. **b**, Plot of the head (blue) and body (green) pitch angles over time, when the simulated insect attempts to have a lateral ventral flow of *ω*_x_ = 0.5. Solid lines are estimates, dashed lines the ground truth. **c**, Estimated (solid) and true (dashed) velocity over time. **d**, Estimated and true height over time. **e**, Optic flow over time. **f**, Body and head rate over time. The head makes much smaller corrections. **g**, Observability degree over time.

For the flapping-wing robot experiments, we used a commercially available ‘flapper drone’. Its design is inspired by the ‘DelFly Nimble’ flapping-wing robot^14^. However, the flapper drone is more robust, which facilitates experiments. It is also larger and heavier than the DelFly Nimble, while staying light-weight compared to most quad rotor drones (100 g). The flapping frequency of the flapper drone is roughly 12 Hz. As explained in the main text, the flapper drone is equipped with the CurvACE^40^, a miniature artificial compound eye, which has a broad field of view (180° × 60°) and a high update rate for the optic flow measurements (200 Hz). Extended Data Fig. 5 shows the experimental setup for the flapper drone, which uses the BitCraze open-source autopilot software. We adapted the flapper drone hardware to include the CurvACE, sending its outputs (four optic flow vectors) to the BitCraze autopilot board. Extraction of ${\omega }_{y}$ is done by averaging the four flow values in the *y* direction, and scaling it with a constant factor to encode rad s^−1^. We also modified the software to run an EKF based on ${\omega }_{y}$ and gyro measurements in parallel to the standard complementary filter, for estimating $\phi$. By contrast to the quad rotor experiments, the outer loop control is performed by a human pilot, providing desired attitude angles and thrust commands. A basic PID controller serves as inner loop controller to reach the desired attitude angles. Again, we can switch between the estimated angle determined by the optic-flow-based EKF and by the accelerometer-based complementary filter. One might be tempted to think that the human pilot could be able to fly the flapper drone even if the roll estimates by the EKF are far off from the true roll angles. However, the inner loop control operates at such a fast time scale that this is not possible: good attitude estimates are necessary for successful flight. The moment and thrust commands are mixed and result in commands to the two independently moving wing pairs for executing the roll and thrust commands. Pitch moments are controlled with a servo that determines the dihedral angle, whereas yaw moments are controlled with a servo that twists the wings slightly for thrust vectoring. For details, we refer the reader to Karásek et al.^14^.

## Online content

Any methods, additional references, Nature Research reporting summaries, source data, extended data, supplementary information, acknowledgements, peer review information; details of author contributions and competing interests; and statements of data and code availability are available at 10.1038/s41586-022-05182-2.

## Supplementary information

Supplementary Information Mathematical derivations and analyses supporting the conclusions in the main article. Section I: We start by deriving the formulas for the non-linear observability analysis of the constant-height system with rotation rate control inputs. Section II: Subsequently, a stability proof for the partially unobservable system is presented. In the following sections, we generalize the model to more complex settings. Section III: A constant-height model without rate measurements. Section IV: A varying height model with drag and wind. Section V: A varying height model with thrust bias and optic flow divergence. Section VI: A model taking into account a ground slope. Section VII: A model of flying in generic 3D-structured environments. Section VIII: We report on simulation experiments that verify different aspects of the proof in section II. Section IX: We introduce a model with an independently moving head and body. Section X: We explain how we analysed biological data for comparison with the model from section IX.

Supplementary Video 1 Quad rotor flying with optic-flow-based attitude. A Parrot Bebop 2 quad rotor’s onboard software has been reprogrammed with the Paparazzi open-source autopilot to hover fully autonomously. Initially, it flies with an inner loop control for the attitude that is based on a standard, complementary attitude estimation filter. This filter combines accelerometer with gyro measurements. During this initial part of the experiment the outer loop control already relies on optic flow. The optic flow divergence is used to set thrust commands, the translational optic flow is used to set attitude commands, executed by the inner loop control. Halfway the experiment, the attitude estimation is switched to the proposed optic-flow-based attitude estimation. This estimation combines a constant-height, thrust-vectoring motion model with optic flow and gyro measurements to estimate both pitch and roll angles.

Supplementary Video 2 Flapper drone flying with optic-flow-based attitude. A flapper drone flapping-wing robot has been equipped with an artificial compound eye, CurvACE. The autopilot software of the flapper drone, based on BitCraze’s CrazyFlie code, has been modified to implement the proposed optic-flow-based attitude estimation. The CurvACE has been programmed to send translational optic flow measurements to the autopilot at 200 Hz. At the start of the experiment, the inner loop attitude control relies on a standard complementary attitude estimation filter, combining accelerometer and gyro measurements. The outer loop control in this experiment consists of manual control, with the human pilot commanding desired attitude angles for use by the inner loop controller. After a bit of flight, a switch is made to the proposed optic-flow-based attitude estimation, only for the roll angle in this experiment. The optic-flow-based attitude estimation combines a constant, thrust-vectoring motion model with CurvACE’s optic flow measurements and gyros.

Supplementary Video 3 Quad rotor flying over a tilted slope. The video shows an experiment to verify the robustness of the proposed optic-flow-based attitude estimation to tilted surfaces. A Parrot Bebop 2 drone with onboard Paparazzi open-source autopilot flies over a tilted slope. The autopilot features an optic flow outer loop control with optic flow divergence leading to thrust commands and translational optic flow to attitude commands. The outer loop control attempts to achieve zero optic flow, that is, hover flight. Initially, the inner loop attitude control uses attitude estimates from a standard complementary filter. After a bit of flight, the attitude estimation is changed to the proposed optic-flow-based attitude estimation, combining a motion model with optic flow and gyro measurements to estimate both pitch and roll. The motion model is based on a flat floor assumption. In this experiment, that assumption is violated, as the drone flies over a tilted screen. In the experiment, first the screen’s tilt angle is increased. Then, the screen is dragged to the side.

Supplementary Video 4 Quad rotor flying over 3D structure. This experiment tests the robustness of the proposed optic-flow-based attitude estimation to non-flat surfaces. A Parrot Bebop 2 drone with onboard the open-source autopilot Paparazzi flies over an area with different-sized plastic plants and chairs. The autopilot features an optic flow outer loop control with optic flow divergence leading to thrust commands and translational optic flow to attitude commands. The optic flow observables, that is, divergence and translational flow, are determined by integrating information from the optic flow vectors in the entire bottom camera field of view. The outer loop control follows different optic flow set points over the experiment, starting with zero flow for hover flight. The attitude estimation starts out with a standard complementary filter, and then switches to the proposed optic-flow-based attitude estimation, combining a motion model with optic flow and gyro measurements to estimate both pitch and roll. The motion model is based on a flat floor assumption. In this experiment, that assumption is violated by means of the objects in the flight arena. The drone first hovers over a large plastic plant, with the leaves moving due to the downwash, violating an extra assumption of a static world. Then the drone receives different outer loop references for non-zero translational optic flow, making it move left and right over the 3D scene.

Supplementary Video 5 Quad rotor subjected to 10° roll disturbances. This experiment tests the robustness of the proposed optic-flow-based attitude estimation to large disturbances. A Parrot Bebop 2 drone with onboard open-source autopilot Paparazzi flies with optic flow outer loop control, with optic flow divergence leading to thrust commands and translational optic flow to attitude commands. The outer loop has zero divergence and lateral flow as optic flow references, for hover flight. Initially, the attitude is estimated with a standard complementary attitude estimation filter. Then, the drone switches to the proposed optic-flow-based attitude estimation scheme for both pitch and roll and first hovers. At many times in the video, the experimenter introduces a disturbance to the roll angle by extraneously adding a bias to the attitude command sent to the inner loop attitude controller. So, the drone is hovering and sending a desired angle of zero degrees to the inner loop attitude controller, but this is extraneously changed to 10°. The inner loop controller attempts to satisfy this demand, resulting in an increasing sidewards velocity. The situation is corrected for by the integrator in the outer loop controller, which attempts to cancel the velocity by changing the desired attitude angle, that is, −10° when hovering again. Then, the experimenter introduces a new disturbance by removing the bias, resulting in the opposite motion. Mats were placed on the floor to ensure that the optic flow algorithms functioned correctly also at higher speeds.

Supplementary Video 6 Quad rotor flying over a static slope: varying height model. The video shows an experiment to verify the robustness of the proposed optic-flow-based attitude estimation to tilted surfaces. A Parrot Bebop 2 drone with onboard Paparazzi open-source autopilot flies over a tilted slope. The autopilot features an optic flow outer loop control with optic flow divergence leading to thrust commands and translational optic flow to attitude commands. The outer loop control attempts to achieve zero optic flow, that is, hover flight. Initially, the inner loop attitude control uses attitude estimates from a standard complementary filter. After a bit of flight, the roll angle attitude estimation is changed to the proposed optic-flow-based attitude estimation, combining a motion model with optic flow and gyro measurements. In contrast to previous experiments, the motion model does not assume a constant height. The model uses the estimated commanded thrust to predict height changes. The motion model does still assume a flat floor. In this experiment, that assumption is violated, as the drone flies over a tilted screen. In the experiment, the screen’s tilt angle is increased.

Supplementary Video 7 Quad rotor flying over a moving slope: varying height model. The video shows an experiment to verify the robustness of the proposed optic-flow-based attitude estimation to moving, tilted surfaces. A Parrot Bebop 2 drone with onboard Paparazzi open-source autopilot flies over a tilted slope. The autopilot features an optic flow outer loop control with optic flow divergence leading to thrust commands and translational optic flow to attitude commands. The outer loop control attempts to achieve zero optic flow, that is, hover flight. Initially, the inner loop attitude control uses attitude estimates from a standard complementary filter. After a bit of flight, the roll angle attitude estimation is changed to the proposed optic-flow-based attitude estimation, combining a motion model with optic flow and gyro measurements. The motion model uses the estimated commanded thrust to predict height changes. The motion model does still assume a flat floor. In this experiment, that assumption is violated, as the drone flies over a tilted screen. In the experiment, first the screen’s tilt angle is increased. Then, the screen is dragged to the side, with the drone following.

Supplementary Video 8 Quad rotor flying over 3D structure: varying height model. This experiment tests the robustness of the proposed optic-flow-based attitude estimation to non-flat surfaces when the motion model allows for varying height. A Parrot Bebop 2 drone with onboard the open-source autopilot Paparazzi flies over an area with differently sized plastic plants, flowers, boxes and a chair. The autopilot features an optic flow outer loop control with optic flow divergence leading to thrust commands and translational optic flow to attitude commands. The optic flow observables, that is, divergence and translational flow, are determined by integrating information from the optic flow vectors in the entire bottom camera field of view. The outer loop control follows different optic flow set points over the experiment, starting with zero flow for hover flight. The attitude estimation starts out with a standard complementary filter, and then switches to the proposed optic-flow-based attitude estimation, combining a motion model with optic flow and gyro measurements to estimate roll. The motion model uses the estimated commanded thrust to predict height changes and is based on a flat floor assumption. In this experiment, that assumption is violated by means of the objects in the flight arena. The drone first hovers over the centre of the scene (the chair). Then the drone receives different outer loop references for non-zero translational optic flow, making it move left and right over the 3D scene.

## Data Availability

All data necessary for performing and analysing the experiments is publicly available: the flight data is available at 10.4121/20183399.

## Extended data

are available for this paper at 10.1038/s41586-022-05182-2.

## Code availability

The code to reproduce the theoretical and simulation results and analyse robotic experiments is publicly accessible at 10.4121/20183399. The code to perform flight experiments with the open-source Paparazzi autopilot on the Bebop 2 drone is available at https://github.com/tudelft/paparazzi/releases/tag/v5.17.5_attitude_flow. The code to perform flight experiments with the flapper drone is available at https://github.com/tudelft/crazyflie-firmware/releases/tag/v3.4.0_attitude_flow.
