# Supplementary material for: Accommodating unobservability to control flight attitude with optic flow
Source: Nature. 2022 Oct 19;610(7932):485–90. doi: 10.1038/s41586-022-05182-2 (PMC9581779; doi:10.1038/s41586-022-05182-2)
Supplement: Supplementary file 1 — Mathematical derivations and analyses supporting the conclusions in the main article. Section I: We start by deriving the formulas for the non-linear observability analysis of the constant-height system with rotation rate control inputs. Section II: Subsequently, a stability proof for the partially unobservable system is presented. In the following sections, we generalize the model to more complex settings. Section III: A constant-height model without rate measurements. Section IV: A varying height model with drag and wind. Section V: A varying height model with thrust bias and optic flow divergence. Section VI: A model taking into account a ground slope. Section VII: A model of flying in generic 3D-structured environments. Section VIII: We report on simulation experiments that verify different aspects of the proof in section II. Section IX: We introduce a model with an independently moving head and body. Section X: We explain how we analysed biological data for comparison with the model from section IX. [file 41586_2022_5182_MOESM1_ESM.docx]

Supplementary information

**ACCOMMODATING UNOBSERVABILITY TO CONTROL FLIGHT ATTITUDE**

**WITH OPTIC FLOW**

**G.C.H.E. de Croon^1^, J.J.G. Dupeyroux^1^, C. De Wagter^1^,**

**A. Chatterjee^1^, D.A. Olejnik^1^, F. Ruffier^2^**

1. Micro Air Vehicle laboratory, Control and Simulation, Faculty of Aerospace Engineering, Delft University of Technology, the Netherlands

2. Aix Marseille Univ, CNRS, ISM, Marseille, France.

In the supplementary information we start by deriving the formulas for the nonlinear observability analysis of the constant height system with rotation rate control inputs (SI-I). Subsequently, we present a stability proof for the partially unobservable system (SI-II). In the following sections, we generalize the model to more complex settings: a constant height model without rate measurements (SI-III), a varying height model with drag and wind (SI-IV), a varying height model with thrust bias and optic flow divergence (SI-V), a model taking into account a ground slope (SI-VI), and a model of flying in generic 3d-structured environments (SI-VII). In SI-VIII we report on simulation experiments that verify different aspects of the proof in SI-II. Moreover, in SI-IX, we introduce a model with an independently moving head and body. Finally, in SI-X we explain how we analyzed biological data for comparison with the model from SI-IX.

## **SI-I: Nonlinear Observability Analysis Constant Height Model**

We will analyze the observability of the states given optic flow measurements when the altitude is constant. We recall that in this case the observation equation is:

|  | $\omega_{y}=-\frac{v_{B}}{Z_{B}}+p=-\frac{{cos}^{2}\left( \varphi\right)v_{I}}{Z_{I}}+p$ | (Eq. 1) |
| --- | --- | --- |

Moreover, the state is defined as: $\vec{\boldsymbol{x}}=\left[ v_{I},\varphi,Z_{I} \right]$, with as control input $u=p$. This leads to the state update equation:

|  | $f(\vec{\boldsymbol{x}},u)=\left[ \begin{matrix} \dot{v_{I}} \\ \dot{\varphi} \\ \dot{Z_{I}} \end{matrix} \right]=\left[ \begin{matrix} g tan\left( \varphi\right) \\ p \\ 0 \end{matrix} \right]$ | (Eq. 2) |
| --- | --- | --- |

Please note that while the motion model assumes $Z_{I}$ to be constant, it will be estimated as part of the state. Hence, the state estimate of $Z_{I}$ can vary over time.

Furthermore, we take as a single observation the flow in y-direction for constant altitude, as this corresponds to the measurement available to many current drones: $y=\omega_{y}=h(\vec{\boldsymbol{x}})$ (Eq. 1). Since we have access to this observation, we can also track it over time and determine the time derivatives. This will form the start of the nonlinear observation analysis, which is based on taking subsequent Lie derivatives $\mathcal{L}_{f}^{.}h$:

|  | $\dot{y}=\frac{\partial y}{\partial t}=\mathcal{L}_{f}^{1}h=\frac{\partial y}{\partial\vec{\boldsymbol{x}}}\frac{\partial\vec{\boldsymbol{x}}}{\partial t}=\frac{\left( 2pv_{I}-g \right)sin\left( 2\varphi\right)}{{2 Z}_{I}}$ | (Eq. 3) |
| --- | --- | --- |

Further deriving over time gives:

|  | $\ddot{y}=\mathcal{L}_{f}^{2}h=\frac{{\partial\mathcal{L}}_{f}^{1}h}{\partial\vec{\boldsymbol{x}}}\frac{\partial\vec{\boldsymbol{x}}}{\partial t}=p\frac{2 p v_{I} cos\left( 2\varphi\right) + g - 2 g cos\left( 2\varphi\right)}{Z_{I}}$ | (Eq. 4) |
| --- | --- | --- |

This leads to the following equation for the observation and its time derivatives, termed the “observability mapping”^1^:

|  | $H\left( \vec{\boldsymbol{x}} \right)=\left[ \begin{matrix} h \\ \mathcal{L}_{f}^{1}h \\ \mathcal{L}_{f}^{2}h \end{matrix} \right]=\left[ \begin{matrix} y \\ \dot{y} \\ \ddot{y} \end{matrix} \right]=\left[ \begin{matrix} -\frac{{cos}^{2}\left( \varphi\right)v_{I}}{Z_{I}}+p \\ \frac{\left( 2pv_{I}-g \right)sin\left( 2\varphi\right)}{{2 Z}_{I}} \\ p\frac{2\left( pv_{I}-g \right)cos\left( 2\varphi\right)+g}{Z_{I}} \end{matrix} \right]$ | (Eq. 5) |
| --- | --- | --- |

**Local, weak observability analysis**

The common way to proceed with (nonlinear) observability analysis is then to perform a local analysis by differentiating $H\left( \vec{\boldsymbol{x}} \right)$ with respect to the state to obtain the observability matrix $\mathcal{O}$:

$$\mathcal{O =}\frac{\partial H\left( \vec{\boldsymbol{x}} \right)}{\partial\vec{\boldsymbol{x}}}=\left[ \begin{matrix} \frac{\partial y}{\partial\vec{\boldsymbol{x}}} & \frac{\partial\dot{y}}{\partial\vec{\boldsymbol{x}}} & \frac{\partial\ddot{y}}{\partial\vec{\boldsymbol{x}}} \end{matrix} \right]=\left[ \begin{matrix} -\frac{{cos}^{2}\left( \varphi\right)}{Z_{I}} & \frac{p sin\left( 2\varphi\right)}{Z_{I}} & \frac{2 p^{2} cos\left( 2\varphi\right)}{Z_{I}} \\ \frac{sin\left( 2\varphi\right)v_{I}}{Z_{I}} & \frac{\left( 2pv_{I}-g \right)cos\left( 2\varphi\right)}{Z_{I}} & 4p\frac{\left( g-pv_{I} \right)sin\left( 2\varphi\right)}{Z_{I}} \\ \frac{{cos}^{2}\left( \varphi\right)v_{I}}{{Z_{I}}^{2}} & \frac{\left( g-2pv_{I} \right)sin\left( 2\varphi\right)}{{{2Z}_{I}}^{2}} & -p\frac{2\left( pv_{I}-g \right)cos\left( 2\varphi\right)+g}{{Z_{I}}^{2}} \end{matrix} \right]$$

(Eq. 6)

If the observability matrix has full rank, the system is said to be locally, weakly observable. Indeed, given the changes in the observable vector ($y$ and its time derivatives), and the observability matrix, we can deduce the change in state:

|  | $\mathcal{O}^{T} d\vec{\boldsymbol{x}} =\left[ \begin{matrix} dy \\ d\dot{y} \\ d\ddot{y} \end{matrix} \right]$ | (Eq. 7) |
| --- | --- | --- |
|  | $d\vec{\boldsymbol{x}} =\mathcal{O}^{-T}\left[ \begin{matrix} dy \\ d\dot{y} \\ d\ddot{y} \end{matrix} \right]$ | (Eq. 8) |

The inversion of $\mathcal{O}$ is only possible if it is full rank, in which case there is a unique solution to $d\vec{\boldsymbol{x}}$. Neither the rows nor the columns of $\mathcal{O}$ are linearly dependent, and there is no row or column of zeroes. Hence, there exist conditions in which the matrix system $\mathcal{O}$ is full rank and in which the state is locally, weakly observable.

However, there are also conditions under which the system becomes locally, weakly unobservable. For instance, the system becomes unobservable when the rotation rate is set to zero, i.e., $p=0$:

| $\mathcal{O}^{'}=\left[ \begin{matrix} -\frac{{cos}^{2}\left( \varphi\right)}{Z_{I}} & 0 & 0 \\ \frac{sin\left( 2\varphi\right)v_{I}}{Z_{I}} & \frac{- g cos\left( 2\varphi\right)}{Z_{I}} & 0 \\ \frac{{cos}^{2}\left( \varphi\right)v_{I}}{{Z_{I}}^{2}} & \frac{g sin\left( 2\varphi\right)}{{{2Z}_{I}}^{2}} & 0 \end{matrix} \right]$ | (Eq. 9) |
| --- | --- |

where $\mathcal{O}^{'}$ has rank 2 due to the column of zeros.

One might expect that setting the inertial velocity to zero also results in local unobservability. However, assuming that $v_{I}=0$ results in:

|  | $\mathcal{O}^{'}=\left[ \begin{matrix} -\frac{{cos}^{2}\left( \varphi\right)}{Z_{I}} & \frac{p sin\left( 2\varphi\right)}{Z_{I}} & \frac{2 p^{2} cos\left( 2\varphi\right)}{Z_{I}} \\ 0 & \frac{- g cos\left( 2\varphi\right)}{Z_{I}} & 4p\frac{g sin\left( 2\varphi\right)}{Z_{I}} \\ 0 & \frac{g sin\left( 2\varphi\right)}{{{2Z}_{I}}^{2}} & -p\frac{-2g cos\left( 2\varphi\right) + g}{{Z_{I}}^{2}} \end{matrix} \right],$ | (Eq. 10) |
| --- | --- | --- |

which can still be full rank. Additionally setting $\varphi=0$, we get:

|  | $\mathcal{O}^{''}=\left[ \begin{matrix} -\frac{1}{Z_{I}} & 0 & \frac{2 p^{2}}{Z_{I}} \\ 0 & \frac{- g}{Z_{I}} & 0 \\ 0 & 0 & \frac{p g}{{Z_{I}}^{2}} \end{matrix} \right]$, | (Eq. 11) |
| --- | --- | --- |

which can also still be full rank. This implies that hovering per se does not induce local unobservability, as long as the rate *p* is not 0. Of course, a perfect hover also means that the rate is 0, which then leads to an unobservable condition. The other important insight from this is that in this model the hover state is not special – any condition becomes unobservable when *p* = 0. So also when the drone attempts to translate with a specific optic flow value, the state will become unobservable when that value is reached.

In order to find all conditions in which the matrix $\mathcal{O}$ is not full rank, we solve for its determinant being zero:

|  | $\left\vert\mathcal{O} \right\vert=-\frac{g p \left( \frac{\cos\left( 2 \phi\right)}{2}+\frac{1}{2} \right) \left( g \cos\left( 2 \phi\right)-2 g+2 p v \cos\left( 2 \phi\right) \right)}{{Z_{I}}^{4}}=0$ | (Eq. 12) |
| --- | --- | --- |

The formula of the determinant immediately shows three interesting cases in which the state will become unobservable. First, there is the known case of $p=0$. Second, the determinant becomes smaller as $z_{I}$ becomes higher, converging to zero when it goes to infinite: $\lim_{z_{I}\to\infty} \left| \mathcal{O} \right|=0$. This makes sense, because at large heights, the translational optic flow becomes negligibly small. This drastically reduces the sensitivity of the optic flow measurement to translational motion, and it will only still measure rotational flow. Third, a zero-gravity environment, i.e., $g=0$, would also make the determinant zero and hence the state unobservable for this constant height model.

We now enumerate the additional solutions to $\left| \mathcal{O} \right|=0$, found by solving the above equation with the MATLAB symbolic toolbox (and verified numerically). Specifically, there is one other solution for $p$:

|  | $p=\frac{2 g-g \cos\left( 2 \phi\right)}{2 v \cos\left( 2 \phi\right)}$ | (Eq. 13) |
| --- | --- | --- |

The solutions for $\varphi$ are:

|  | $\varphi=\frac{1}{2}\pi$ | (Eq. 14) |
| --- | --- | --- |
|  | $\varphi=-\frac{\text{acos}\left( \frac{2 g}{g+2 p v} \right)}{2}$ | (Eq. 15) |
|  | $\varphi=\frac{\text{acos}\left( \frac{2 g}{g+2 p v} \right)}{2}$ | (Eq. 16) |

, with as conditions that:

|  | $g^{2}\leq4 p^{2} v^{2}\wedge\frac{4 p^{2} v^{2}}{3}\leq3 \left( g+\frac{4 p v}{3} \right)^{2}$ | (Eq. 17) |
| --- | --- | --- |

And the solution for $v_{I}$ is:

|  | $v_{I}=\frac{2 g-g \cos\left( 2 \phi\right)}{2 p \cos\left( 2 \phi\right)}$ | (Eq. 18) |
| --- | --- | --- |

Studying these solutions shows that they are quite unlikely to occur, as they often require quite high rates, angles, or velocities. The solution $\varphi=\frac{1}{2}\pi$ is even an invalid condition, since we restrict the angle to $(-\frac{1}{2}\pi,\frac{1}{2}\pi)$. This is due to the flat floor assumption and the sensor not seeing any world point anymore outside this interval (see MM-I). Even more important, though, is that all these solutions are transient when $p\neq0$. A nonzero rate will change the angle $\varphi$, and a nonzero $\varphi$ will in turn change the velocity $v_{I}$. Consequently, the equalities above will only occur for a very short time instant. Hence, from here on we will consider $p=0$ as the main condition for which the state of this model is unobservable.

Finally, we have performed a numerical verification, determining the rank of the observability matrix for 10000 samples in the state intervals: $v_{I}\in[-10,10]$ m/s, $\varphi\in[-60,60]$ ^o^, $z\in(0,20]$ m, and $p\in[-90,90]$ ^o^/s. All of these samples represented conditions in which the state is observable, which is understandable given that the chance of an exactly zero rate is exceedingly small for such random sampling. Furthermore, we determined the “observability degree” (see Eq. 35) of these samples, resulting in a mean degree of $d(\mathcal{O})=0.54$ with a standard deviation of $\sigma_{d}=0.16$.

**Local observability analysis**

It is also possible to analyze the stronger property of local observability^2^. Here, we investigate local observability by taking the observability mapping (Eq. 5) and solving it as a set of nonlinear equations. This analysis considers $y$ and its time derivatives as knowns, since these can actually be observed or derived over time by the drone. Hence, in the formulas that follow, we will set $\left[ \begin{matrix} y & \dot{y} & \ddot{y} \end{matrix} \right]=\left[ \begin{matrix} C_{1} & C_{2} & C_{3} \end{matrix} \right]$, to illustrate that these are now known constants. We will solve the observability mapping by subsequent substitution. We start with:

|  | $C_{1}=-\frac{{cos}^{2}\left( \varphi\right)v_{I}}{Z_{I}}+p$ | (Eq. 19) |
| --- | --- | --- |

which can be transformed to:

|  | $v_{I}=-\frac{\left( C_{1}-p \right)Z_{I}}{{cos}^{2}\left( \varphi\right)}$ | (Eq. 20) |
| --- | --- | --- |

where it should be noted that $\varphi$ is in the interval $\left( -90^{\circ}, 90^{\circ} \right)$, i.e., with the limits excluded. This is logical given our definitions of the variables and optic flow measurements (Fig. 4). This expression for $v_{I}$ can then be inserted into the next equation, of $\dot{y}$, and we can solve for the next variable:

|  | $C_{2}=-\frac{\sin\left( 2 \varphi\right) \left( g+\frac{2 p Z_{I} \left( C_{1}-p \right)}{\cos^{2} \left( \varphi\right)} \right)}{2 Z_{I}}$ | (Eq. 21) |
| --- | --- | --- |

, which can be solved for $Z_{I}$ as follows:

|  | $Z_{I}=-\frac{g}{\frac{2 C_{2}}{\sin\left( 2 \varphi\right)}+\frac{2 p \left( C_{1}-p \right)}{\cos^{2} \left( \varphi\right)}}$ | (Eq. 22) |
| --- | --- | --- |

showing that we cannot find a solution for $\varphi=0$, as it would lead to a division by zero. Now we can substitute both $Z_{I}$ and $v_{I}$ into the third equation of $\ddot{y}$:

$$C_{3}=-\frac{p \left( \frac{2 C_{2}}{\sin\left( 2 \varphi\right)}+\frac{2 p \left( C_{1}-p \right)}{\cos^{2} \left( \varphi\right)} \right) \left( g-2 g \cos\left( 2 \varphi\right)+\frac{2 g p \cos\left( 2 \varphi\right) \left( C_{1}-p \right)}{\cos^{2} \left( \varphi\right) \left( \frac{2 C_{2}}{\sin\left( 2 \varphi\right)}+\frac{2 p \left( C_{1}-p \right)}{\cos^{2} \left( \varphi\right)} \right)} \right)}{g}$$

(Eq. 23)

which we then need to solve for $\varphi$. We solve the last equation for $\varphi$ with the help of automated tools, specifically MATLAB’s symbolic “solve” function. The solutions are expressed as:

$$\varphi=2 \text{atan}\left( C_{2} p k^{6}+2 C_{3} k^{5}-15 C_{2} p k^{4}+32 C_{1} p^{2} k^{3}-4 C_{3} k^{3}-32 p^{3} k^{3}+15 C_{2} p k^{2}+2 C_{3} k-C_{2} p \right)$$

(Eq. 24)

for all six roots of the polynomial in $k$. Hence, there are six different solutions for $\varphi$. However, these are not all feasible. Some solutions involve a $\varphi$ that is too large, others result in an impossible negative height $Z_{I}$ or a height with an imaginary component.

Please note that there are two cases in which finding such a solution is not possible: $p=0$ and $\varphi=0$. Setting $p=0$ leads to $\ddot{y}=0$. This means that we are left with two nonlinear equations with three unknown states. Hence, as expected, also for the stronger property of local observability, the state becomes unobservable when there is no rotation rate. Setting $\varphi=0$ leads to divisions by zero, e.g., in finding the solution to $Z_{I}$. For a more intuitive understanding of why these states may be problematic, please realize that in the constant height model $p=0$ means that the (horizontal) jerk is zero and $\varphi=0$ means that the (horizontal) acceleration is zero. If both are zero the system has a constant velocity, which leads to an optic flow that is constant over time, $\omega_{y}=-\frac{v_{B}}{Z_{B}}=C=-\frac{{a v}_{B}}{a Z_{B}}$, with $C$ the constant flow value and the equation being valid for any scaling factor $a\in\left( 0,\infty\right)$. This represents the well-known constant optic flow case in which velocity and height cannot be disentangled.

We verified the above solution procedure numerically, by randomly setting a ground truth roll, rate, velocity and height in the intervals $\varphi\epsilon\left( -90^{\circ}, 90^{\circ} \right), p \epsilon\left[ -90,90 \right]^{\circ}/s, v \epsilon\left[ -5,5 \right]$ m/s, and $z \epsilon\left[ 0,20 \right]$ m, respectively, where “[” and “]” mean that the border values are included. We then calculated the observation vector $\left[ \begin{matrix} C_{1} & C_{2} & C_{3} \end{matrix} \right]$ and determined the solution with the formulas above. When we: (i) exclude $p=0$ and $\varphi=0$ as ground truth states, (ii) limit the estimated attitude angle $\varphi$ to the interval $\left( -90^{\circ}, 90^{\circ} \right)$, (iii) only accept solutions for which the estimated $Z_{I}>0$, (iv) reject solutions with an imaginary components, and (v) determine $y$ for the solution and only accept it when equal to $C_{4}$, then we always end up with a single solution that matches the ground truth state. Condition (v), which in this model relates to the snap of the trajectory, is only rarely needed to differentiate between alternate solutions. The solutions it rejects are higher roots corresponding to an alternative state with a large angle and very high velocity (e.g., 89^o^ and 200 m/s) that still lead to an identical observation vector, but only until $C_{3}$.

In conclusion, the state is not strictly locally observable, as $p=0$ and $\varphi=0$ lead to a range of solutions. Moreover, mathematically, we did not find a unique solution to the three nonlinear equations of the three-dimensional observation vector. Still, our numerical analysis indicates that the observation vector $\left[ \begin{matrix} y & \dot{y} & \begin{matrix} \ddot{y} & y \end{matrix} \end{matrix} \right]=\left[ \begin{matrix} C_{1} & C_{2} & \begin{matrix} C_{3} & C_{4} \end{matrix} \end{matrix} \right]$ has a one-to-one mapping to the state for the investigated intervals of ground-truth states. This suggests that, excluding $p=0$ and $\varphi=0$, in these intervals the state is observable, and there are no local minima for state estimation filters to get stuck in.

Since $p$ is a control input and can be set nonzero, and $\varphi=0$ will be a transient state for nonzero $p$, the numerical findings suggest that the system is likely globally observable^2^, that is, a weaker property than local observability in which longer times are allowed to differentiate between states. Finally, we emphasize that the practical use of the solution formulas given above for direct state estimation is limited, since the derivatives of $y$ will be noisy.

# SI-II: Stability proof of a partially unobservable system

**Theorem 1:**

*Assume the constant altitude translational optic flow model and the presence of the following factors: measurement noise, actuation noise, and external disturbances. Given these assumptions, an attitude controller will intermittently achieve a desired attitude* $\varphi^{*}$ *despite the fact that achieving this control goal leads to unobservability of the state.*

“Intermittently” means that the state will continuously alternate between converging to the desired states and – after reaching them – deviating from them again. Furthermore, we do not assume a specific noise distribution, other than that it is not a Dirac function at 0 (i.e., no noise).

**Proof**

Our proof consists of two parts. The first part shows that when the system is observable, the controller can stabilize the system at a desired attitude angle. Since the control input is the attitude rate, it goes to zero when the setpoint is reached. Then the system becomes unobservable. The second part shows that when the system is unobservable, either the system or the state estimate will drift from its desired, true state, leading to actions of the rate controller, which make the system observable again.

**Part I: Stable control will lead the observable system to the desired attitude, with zero rate**

When the state is observable, we can control the drone to reach a desired attitude. We will prove this with a Lyapunov stability analysis. This analysis shows how a state variable of interest (in our case the attitude error) evolves over time. Specifically, we choose a Lyapunov function of the attitude error that is strictly positive. Subsequently, we prove that there is a control law that will result in a continuous decrease of this strictly positive function, driving the function and hence the attitude error to zero.

As a reminder, the state variables are: $\vec{\boldsymbol{x}}=\left[ v_{I},\varphi,Z_{I} \right]$. The state update equation is:

|  | $\left[ \begin{matrix} \dot{v_{I}} \\ \dot{\varphi} \\ \dot{Z_{I}} \end{matrix} \right]=\left[ \begin{matrix} g tan\left( \varphi\right) \\ p \\ 0 \end{matrix} \right]$ | (Eq. 25) |
| --- | --- | --- |

As a Lyapunov function, we will take:

|  | $V=\left( \varphi-\varphi^{*} \right)^{2}$ | (Eq. 26) |
| --- | --- | --- |

We then determine:

|  | $\dot{V}=\frac{\partial V}{\partial t}=\frac{\partial V}{\partial\vec{\boldsymbol{x}}}\frac{\partial\vec{\boldsymbol{x}}}{\partial t}=\left[ \begin{matrix} 0 & 2\left( \varphi-\varphi^{*} \right) & 0 \end{matrix} \right]\left[ \begin{matrix} g tan\left( \varphi\right) \\ p \\ 0 \end{matrix} \right]=2p\left( \varphi-\varphi^{*} \right)$ | (Eq. 27) |
| --- | --- | --- |

This shows that a simple proportional gain controller can drive the error with the desired angle to zero. In particular, the following control law can be used:

|  | $p=-K\left( \varphi-\varphi^{*} \right),K>0,$ | (Eq. 28) |
| --- | --- | --- |

where $p$ is the commanded rate, and $K$ a positive control gain. Inserting Eq. 28 into Eq. 27 results in:

|  | $\frac{\partial V}{\partial t}=-2K\left( \varphi-\varphi^{*} \right)^{2}<0.$ | (Eq. 29) |
| --- | --- | --- |

The conclusion that $\dot{V}<0$ follows from both $K$ and $\left( \varphi-\varphi^{*} \right)^{2}$ being positive. Since the Lyapunov function always decreases over time and is bounded to be positive, the system will eventually lead to $V=\left( \varphi-\varphi^{*} \right)^{2}=0$ . This implies that the roll attitude angle $\varphi$ will be equal to the desired attitude $\varphi^{*}$.

In conclusion, the straightforward control law used in the Lyapunov function above can bring the system to a desired angle when the system is observable. The problem is that the same control law will lead to $p=0$ when the desired attitude angle is reached (Eq. 28). This makes the system *unobservable*.

**Part II: Unobservable conditions always lead to observable conditions**

When $p=0$ the state becomes unobservable. This term sounds rather ominous, as if in this condition the drone’s state estimate can wander to any position in the state space. This is not true in general. In the case of the studied system, when the state is unobservable there is no longer a single point but a manifold in the state space for which the observation vector $H\left( \vec{\boldsymbol{x}} \right)$ (Eq. 5) has the same values. Hence, states on this “unobservability manifold” are indistinguishable.

However, state estimation filters will still perform corrections of the state estimate based on the observation vector to reduce the error between the current observation vector $\hat{H}(\vec{\boldsymbol{x}})$ and the expected observation vector at the current estimated state $H\left( \hat{\boldsymbol{x}} \right)$. There is a partial gradient that can be followed to update the state in order to reduce the error $\hat{H}-H\left( \hat{\boldsymbol{x}} \right)$, as can be seen for example for $O^{'}=\left. \frac{\partial H\left( \vec{\boldsymbol{x}} \right)}{\partial\vec{\boldsymbol{x}}} \right|_{p=0}$. State estimation filters like the Kalman filter will follow this partial gradient. However, as long as the state is not observable, the gradient to reduce this error is not unique and will not converge to a single point but to another unobservability manifold.

Given the expression of the observation vector $H\left( \vec{\boldsymbol{x}} \right)$ in Eq. 5, we can find an algebraic expression for the manifold. Since $p=0$, $\ddot{y}=0$. Moreover, based on the equation for $y$:

|  | $y=-\frac{{cos}^{2}\left( \varphi\right)v_{I}}{Z_{I}}$ | (Eq. 30) |
| --- | --- | --- |

or:

|  | $v_{I}=-\frac{C_{1}Z_{I}}{{cos}^{2}\left( \varphi\right)}$ | (Eq. 31) |
| --- | --- | --- |

where $y$ has been replaced with $C_{1}$ to indicate that it is a constant in this equation. Furthermore, based on the equation of $\dot{y}$:

|  | $\dot{y}=\frac{-g sin\left( 2\varphi\right)}{{2 Z}_{I}}$ | (Eq. 32) |
| --- | --- | --- |

which leads to the relation:

|  | $Z_{I}=-\frac{g}{{2C}_{2}} sin\left( 2\varphi\right)$ | (Eq. 33) |
| --- | --- | --- |

where $\dot{y}$ has been replaced with the specific constant $C_{2}$. Hence, if $p=0$, the state estimate will converge to the manifold defined by Eq. 31 and 33. When $\dot{y}=C_{2}=0$, the manifold is a surface. If it is nonzero, the manifold is a curve.

When the drone enters the unobservable state, the state estimate will not automatically start wandering over the manifold. In fact, in a perfect world, without factors such as measurement noise, actuation noise, and external disturbances, the observation vector will match that associated with the currently estimated, desired state and the state will remain in the desired state indefinitely. Hence, it could be said that the unobservability is inconsequential in such a perfect world.

Of course, in a real-world system, the three factors mentioned above are present almost all the time. If any of them leads to a nonzero rate, $p\neq0$, the state becomes observable again, bringing us back to part I of this proof. As it turns out, all three effects lead to nonzero rates, so any one of them will induce observability.

*(1) Measurement noise*

Measurement noise leads the drone to believe that it is in a state that it is not. Any noise will take the observation vector away from the unobservability manifold, since the manifold is defined as (and hence contains) all states that would give the same observation vector as in the desired state. Let us look at the two measurements in the constant altitude model.

First, the rotation rate measurements are considered control inputs. If there is noise on the rotation rate, this will lead to a prediction of the attitude that is away from the desired attitude. This prediction will be partially, but not entirely corrected by the optic flow measurement. Hence, noise on the rate measurement will lead to an estimated attitude that no longer matches the desired attitude. This will lead to a “corrective” action, in which the drone rotates towards the desired state. Although this action will actually lead the drone away from the desired state, it will make the state observable again, since $p\neq0$.

Please note that we could stop the proof here, as noise on the gyro measurements is sufficient to induce observability. For completeness, we will discuss the other sensor measurement and afterward also actuation noise and external disturbances.

Second, noise in the optic flow measurement will also typically lead to an update of the attitude estimate. The noisy measurement will result in an observation vector that is different from the one in the current, desired state: $\hat{H}\neq H(\boldsymbol{x}^{*})$. This will lead to an update of the estimated state. Since the observation vector has a gradient to all three states (see Eq. 6), typically all of them will be updated, albeit to different extents. It is theoretically possible that only the velocity and / or height are updated, when $\frac{\partial H}{\partial v_{I}}$ and / or $\frac{\partial H}{\partial Z_{I}}$ are nonzero while $\frac{\partial H}{\partial\varphi}=0$. There is indeed such an observation vector, which can be found by finding the solution to $\frac{\partial H}{\partial\varphi}=0$, i.e., setting the second row of Eq. 6 to zero, assuming $p=0$ (as in Eq. 9). The solution to this is $v=0$ and $\varphi=\frac{1}{4}\pi$, with any $z>0$, corresponding to $\hat{H}=\left[ 0,\frac{-g}{2Z_{I}},0 \right]^{T}$. Noise leading to this observation vector will be extremely rare and can only be instantaneous. Hence, normally the attitude angle will be updated, which means that it no longer corresponds to the desired one. This again leads to a falsely corrective action, inducing $p\neq0$.

*(2) Actuation noise*

Actuation noise will induce changes to either the moments or the thrust. First, any nonzero moment will induce a nonzero rate, $p\neq0$, implying that the state is observable again.

Second, noise in the thrust will lead to a decrease or increase in vertical acceleration. If $\varphi=0$, this will remain unnoticed, since the resulting changes to vertical velocity are not observed by the lateral flow. This is a non-trivial case in which the state can drift over the manifold along the $z_{I}$ coordinate. One way to solve this is to include optic flow divergence in the observations, which is done in the robotic experiments. If $\varphi\neq0$, thrust changes will also lead to horizontal acceleration changes. Since the acceleration then no longer corresponds to that of $\varphi^{*}$, i.e., $\hat{H}\neq H(\boldsymbol{x}^{*})$, the attitude estimate will get (wrongly) updated. This will lead to falsely corrective actions.

*(3) External disturbances*

The effects of external disturbances are very similar to those of actuation noise. If there is an external disturbance that creates a moment, then this will result in a nonzero attitude rate, and hence observability. If there is an external disturbance like a wind gust that induces a force on the body, then any resulting horizontal acceleration will be attributed to an attitude change, leading to falsely corrective actions. The resulting vertical accelerations will typically at least partially be attributed to attitude changes, except when $\varphi=0$. Hence, most horizontal and vertical accelerations due to external disturbances will lead to falsely corrective actions, making the state observable again.

$$∎$$

**Discussion and verification of the proof**

The proof given above has a few characteristics that merit further discussion. First, observability is treated as a binary property. Although this does correspond to its formal definition, in a real system the estimated state will converge to the true state over time and will do so quicker when the system is better observable. Krener and Ide^3^ introduced the local estimation condition number, $\kappa$, to measure the local observability of a non-linear system. It is the ratio of the largest singular value of the local observability matrix with the smallest one:

|  | $\kappa(\mathcal{O})=\frac{s_{max}(\mathcal{O})}{s_{min}(\mathcal{O})}$ | (Eq. 34) |
| --- | --- | --- |

where $s_{max}(\mathcal{O})$ and $s_{min}(\mathcal{O})$ represent the maximum and minimum local singular values of the observability matrix $\mathcal{O}$ (Eq. 6). High values of $\kappa$ indicate that the observability matrix is ill-conditioned, with $\kappa$ of a singular matrix being infinity, and low values of $\kappa$ indicate that the matrix is well-conditioned, with $\kappa$ of an identity matrix being 1. In this article, we have adopted the reciprocal of the log of $\kappa$ as a measure of the degree of observability, $d$, of the system:

|  | $d(\mathcal{O})=\frac{1}{log(\kappa(\mathcal{O}))}$ | (Eq. 35) |
| --- | --- | --- |

This has been done to have the values of d vary between 0 and 1, with higher values of d signifying a higher observability of the system. As shown in Figure 1d&e in the main article, the state is poorly observable for a small region around $p=0$. Hence, slightly off from the desired state, the state can still be barely observable, so that the estimated state may remain faulty and falsely corrective actions may persist in this region. In other words, instead of a manifold in the state space, approximate unobservability may occupy a *volume* in state space around the pure unobservability manifold. This does not essentially change the proof. It just means that the noise / disturbances / false corrective actions will have to accumulate longer before being large enough for the state to converge enough towards the true state to allow for truly corrective actions. This will be reflected in larger and slower oscillations around the desired state. Whether such a real system is stable will depend on its observability and controllability characteristics. Moreover, we want to stress that observability of the system does not automatically imply that a state estimator such as an Extended Kalman Filter (EKF) will converge to the correct state.

Second, part I of the proof consists of a Lyapunov stability analysis, only considering (i) asymptotic stability (potentially taking a very long time to reach the desired state) for (ii) a delay-less control system. Although these are indeed limitations of the proof given above, attitude control with basic PID-controllers is applied widely and successfully. Desired attitudes with such controllers are typically attained quickly for real-world flying systems with noise and delay. Hence, we do not see this as an important limitation. One may also wonder how an outer loop controller such as a velocity controller or optic flow controller would influence the proof. For such outer loop controllers, part I would need to be extended, which could be done with a nested Lyapunov analysis assuming time scale separation. Part II would be extended to include the falsely active corrections caused by the outer loop controller, for instance, when the optic flow controller changes $\varphi^{*}$ in reaction to noise on the flow measurement. Hence, outer loop controllers further increase the probability for taking actions and inducing observability.

Finally, in SI-VIII, we verify individual elements of the theoretical proof with simulation experiments. We show what happens when there are no real-world factors in play, and piece apart the effects of sensor noise, actuation noise, and disturbances. We also investigate “malicious noise” that makes the estimated state wander over the unobservable manifold.

## **SI-III: Constant height model without rate measurements**

The constant height model from the main article considers the rate p as a control input. However, real-world systems like drones and flying insects do not control attitude rate directly. Instead, by varying rotor speeds or wing flapping amplitudes, they generate moments. Hence, the model explained in the main manuscript can be seen as having a low-level rate controller that uses rate measurements (e.g., from gyros or halteres) to generate the appropriate moments.

Here we investigate a constant altitude, changing attitude model in which it is assumed that the control input is a moment. Moreover, the attitude rate p is estimated as a state instead of measured or known. This corresponds to the case of a robot without gyros (or insect without halteres) that additionally does not separate the rotational and translational optic flow measurements to get a rate measurement. The system equations are:

|  | $\left[ \begin{matrix} \dot{v_{I}} \\ \begin{matrix} \dot{\varphi} \\ \dot{p} \end{matrix} \\ \dot{Z_{I}} \end{matrix} \right]=\left[ \begin{matrix} g tan\left( \varphi\right) \\ \begin{matrix} p \\ M/I \end{matrix} \\ 0 \end{matrix} \right]$ | (Eq. 36) |
| --- | --- | --- |

where $p$ is now a state, $M$ is the moment and $I$ is the moment of inertia. Assuming that only the ventral lateral flow is observed, $\omega_{y}$, we arrive at the following observability mapping, using $v,z$ as shorthands for $v_{I},Z_{I}$:

|  | $H\left( \vec{\boldsymbol{x}} \right)=\left[ \begin{matrix} p-\frac{v \cos^{2} \left( \varphi\right)}{z} \\ \frac{M z-\frac{I g \sin\left( 2 \varphi\right)}{2}+I p v \sin\left( 2 \varphi\right)}{I z} \\ \begin{matrix} \frac{M v \sin\left( 2 \varphi\right)+I g p+2 I p^{2} v \cos\left( 2 \varphi\right)-2 I g p \cos\left( 2 \varphi\right)}{I z} \\ H_{4} \end{matrix} \end{matrix} \right]$ | (Eq. 37) |
| --- | --- | --- |

where:

$$H_{4}=$$

$$-\frac{8 I v \sin\left( \varphi\right) p^{3} \cos^{2} \left( \varphi\right)-12 I g \sin\left( \varphi\right) p^{2} \cos^{2} \left( \varphi\right)+2 I g \sin\left( \varphi\right) p^{2}-12 M v p \cos^{3} \left( \varphi\right)+6 M v p \cos\left( \varphi\right)+6 M g \cos^{3} \left( \varphi\right)-5 M g \cos\left( \varphi\right)}{I z \cos\left( \varphi\right)}$$

(Eq. 38)

This observability mapping leads to an observability matrix $\mathcal{O}$ that has full rank. We omit $\mathcal{O}$ here, since the equations are too extensive. Also for the analyses of the other models in the sub-sections below, we refrain from showing the full vectors and matrices for this same reason. The reader can check all the findings on nonlinear observability analysis by running the MATLAB code that we used for the analysis. It leverages the symbolic toolbox for determining $H$, $\mathcal{O}$, and $\mathcal{O}$’s rank. Please note that MATLAB’s rank function is not infallible when applied to symbolic matrices. Notably, it may miss equivalences between different trigonometric functions. As an extra verification, as for the system analyzed in SI-I, we sample 10000 states in the same state space interval, but now also with a rotational acceleration from the interval $M/I\in{[-10,10]}^{o}/s^{2}$. Substituting the symbolic variables in the observability matrix with the values of the sampled state resolves the problem that MATLAB has with evaluating the rank of symbolic matrices with trigonometric functions. All samples led to an observability matrix of full rank. Since there can still be numerical round-off errors in MATLAB when a matrix is close to being ill-conditioned, we again also determined the mean and standard deviation of the observability degree (Eq. 35): $\bar{d(\mathcal{O)}}=0.32$, $\sigma_{d}=0.079$, reflecting that the large majority of the evaluated matrices is well away from being ill-conditioned.

Also in this model, in which only the moment is known (or predicted) and optic flow is measured at the center image coordinate, the state is locally, weakly observable. In other words, separate rate measurements, as can be obtained by means of a gyro, are not necessary for observability. This is the model for which the degree of observability and control results are shown in Fig. 1d,f (without rate measurements). In this case, the state becomes unobservable when setting both the moment $M$ and attitude rate $p$ to zero:

|  | ${\mathcal{O}\left. \right\vert}_{p=0,M=0}=\left[ \begin{matrix} -\frac{\cos^{2} \left( \varphi\right)}{z} & 0 & 0 & 0 \\ \frac{v \sin\left( 2 \varphi\right)}{z} & -\frac{g \cos\left( 2 \varphi\right)}{z} & 0 & 0 \\ 1 & \frac{v \sin\left( 2 \varphi\right)}{z} & \frac{g-2 g \cos\left( 2 \varphi\right)}{z} & 0 \\ \frac{v \cos^{2} \left( \varphi\right)}{z^{2}} & \frac{g \sin\left( 2 \varphi\right)}{2 z^{2}} & 0 & 0 \end{matrix} \right]$ | (Eq. 39) |
| --- | --- | --- |

Please note that in the main article this model is compared with the same model but then with rate measurements (Fig. 1e,g). That is, in that case the observations consist of the lateral optic flow and the rotation rate: $\boldsymbol{y}={[\begin{matrix} \omega_{y} & p \end{matrix}]}^{T}$.

## **SI-IV: Varying height model with drag and wind**

One may wonder if the state is still observable when the system is free to move up and down and is subjected to drag and wind. We employ the following model:

|  | $\left[ \begin{matrix} \dot{v} \\ \begin{matrix} \dot{\varphi} \\ \dot{p} \end{matrix} \\ \dot{\begin{matrix} z \\ \begin{matrix} \dot{w} \\ \dot{\begin{matrix} A_{y} \\ \dot{A_{z}} \end{matrix}} \end{matrix} \end{matrix}} \end{matrix} \right]=\left[ \begin{matrix} T sin\left( \varphi\right)/m - S(v-A_{y}) k_{d}{(v-A_{y})}^{2} \\ \begin{matrix} p \\ M/I \end{matrix} \\ \begin{matrix} w \\ \begin{matrix} T cos\left( \varphi\right)/m - g - S(w-A_{z}) k_{d}{(w-A_{z})}^{2} \\ \begin{matrix} 0 \\ 0 \end{matrix} \end{matrix} \end{matrix} \end{matrix} \right]$ | (Eq. 40) |
| --- | --- | --- |

where $T$ is the thrust, $m$ is the mass, $S(\cdot)$ is the sign function, $A$ is the wind (with a $y$ and $z$ component), $k_{d}$ the drag “factor”, and $w$ is the vertical inertial velocity. Moreover, as before, $v$ is the inertial horizontal velocity and $z$ the inertial height. Please note that the drag factor $k_{d}$ captures multiple terms present in the normal drag equation, such as air density, surface area, and the drag coefficient.

Nonlinear observability analysis shows that this varying height model is still locally, weakly, observable, unless we set the thrust to compensate for gravity, the velocities to match the wind, and the moment and rate to zero. This setting corresponds to a condition of a pure hover in this model. We also performed a numerical verification evaluating the observability matrix’s rank for 10000 samples from the state space. The state space intervals are the same as for the previous models, with additionally $A_{y}$, $A_{z}\in[-20,20]$ m/s. The state was locally, weakly observable for all samples, with average observability degree 0.10 and standard deviation 0.018.

The observability of this varying height model is quite surprising, given that the model’s only observation is a single flow vector at the principal axis. We do want to note that this setup should not be used for practical implementation. In order to form the observation vector $H$, the observation $y=\omega_{y}$ has to be differentiated over time six times (so up to $y^{(6)}$). Since y itself is noisy, a filter based on only this single flow measurement will have a difficult time accurately estimating all variables.

The observability of the state will vastly improve by adding more sensor measurements, such as optic flow divergence, here denoted as $\omega_{z}=\frac{w_{B}}{Z_{B}}$, and the rotation rate $p$. For local observability analysis, there are then various options for constituting the observation vector, e.g., $H=[{\omega_{y},\dot{\omega_{y},}\ddot{\omega_{y},}\omega_{z},\dot{\omega_{z}},p]}^{T}$ or $H=[{\omega_{y},\dot{\omega_{y},}\omega_{z},\dot{\omega_{z}},\ddot{\omega_{z},}p]}^{T}$. In any case, it requires fewer differentiations over time, making it more likely that such a filter will work well in practice.

## **SI-V: Varying height model with thrust bias and optic flow divergence**

The robotic experiments reported on in the main article have been performed with the basic constant height model discussed in MM-I and analyzed in SI-I. This model does not estimate the height well when there are substantial height changes (Fig. ED-6b). The height changes only slightly affect the estimation errors of the attitude angles (mean absolute error of $\varphi$, $\theta$ of 0.61, 0.53 vs. 0.43, 0.37 in constant height conditions). Nonetheless, we have also tested a varying height model with drag (but without wind) on the quadrotor (Fig. ED-6c). Here we explain this model that was implemented on the robot.

Initially we employed a basic varying height model with horizontal drag in our experiments, where the thrust was directly predicted from the rotor commands. However, small prediction errors led to large estimation errors of $Z_{I}$. Hence, we finally included a “thrust bias” state variable, which can capture effects such as an inaccurate hover thrust estimate or the thrust diminishing for the same command when the battery voltage drops.

The inclusion of a thrust bias leads to the following model:

|  | $\left[ \begin{matrix} \dot{v} \\ \dot{\varphi} \\ \begin{matrix} \dot{z} \\ \dot{w} \\ \dot{T_{b}} \end{matrix} \end{matrix} \right]=\left[ \begin{matrix} (T-T_{b})sin(\varphi)/m - S(v) k_{d}v^{2} \\ p \\ \begin{matrix} w \\ (T-T_{b})cos(\varphi)/m-g \\ 0 \end{matrix} \end{matrix} \right]$ | (Eq. 41) |
| --- | --- | --- |

With as observation vector:

|  | $\boldsymbol{y=}\left[ \begin{matrix} \omega_{\boldsymbol{y}} \\ \omega_{\boldsymbol{z}} \end{matrix} \right]\boldsymbol{,}$ | (Eq. 42) |
| --- | --- | --- |

i.e., containing both the ventral lateral optic flow and divergence. The rotation rate serves as a known control input in the model but is measured by means of the gyros on the robot. Using $H=[{\omega_{y},\dot{\omega_{y},}\ddot{\omega_{y},}\omega_{z},\dot{\omega_{z}}]}^{T}$ as an observation vector, the system is locally, weakly observable (including the thrust bias). Also here, the formulas for the observability matrix are omitted since they are rather extensive. We perform a numerical verification of the rank for 10,000 samples in the same state space intervals as in SI-I, but now additionally with $T\in[-3g,3g]$ and a thrust bias $T_{b}\in[-0.3g,0.3g]$. The verification reveals that all samples are locally, weakly observable with an average observability degree of 0.32 and standard deviation 0.076. When we set the thrust to compensate gravity and set $v,p,$ and $\varphi$ to zero, the state becomes unobservable.

## **SI-VI: Surface with a slope**

All the models discussed above assumed a flat ground. Here, we study what happens if the surface is sloped. Please note that surface slope could be detected visually by processing the entire optic flow field^4^, but that we assume here that the robot only uses the elementary optic flow measurement at the principal axis: $y=\omega_{y}$.

Figure ED-3c shows the quad rotor when flying over a surface with slope in the $Y_{I}$ direction. This leads to the following model, in which $\alpha$ is the slope angle, and $Z_{I}$ is now the changing height of the drone over the sloped surface:

|  | $f(\vec{\boldsymbol{x}},u)=\left[ \begin{matrix} \dot{v_{I}} \\ \dot{\varphi} \\ \begin{matrix} \dot{Z_{I}} \\ \dot{\alpha} \end{matrix} \end{matrix} \right]=\left[ \begin{matrix} g tan\left( \varphi\right) \\ p \\ \begin{matrix} v_{I} tan\left( \alpha\right) \\ 0 \end{matrix} \end{matrix} \right]$ | (Eq. 43) |
| --- | --- | --- |

Furthermore, the observation equation now becomes:

|  | $\omega_{y}=-\frac{v_{B}}{Z_{B}}+p=-\frac{cos\left( \varphi\right)cos\left( \varphi+\alpha\right)v_{I}}{Z_{I} cos\left( \alpha\right)}+p$ | (Eq. 44) |
| --- | --- | --- |

using $Z_{B}=\frac{cos(\alpha+\varphi)}{Z_{G}}$, $Z_{G}=cos(\alpha)Z_{I}$ and $v_{B}= cos\left( \varphi\right)v_{I}$. The resulting matrix $\mathcal{O}$ is full rank, showing that also this model is locally, weakly observable. Numerical verification with 10000 samples with $\alpha\in[-60,60]^{\circ}$ shows that for all samples the state is locally, weakly observable, with as average observability degree 0.31 with standard deviation 0.13. Hence, even if the vision measurement itself does not disentangle slope from attitude angle, in principle a state estimation filter using this motion model can separate the slope angle from the attitude angle.

## **SI-VII: Optic-flow-based attitude estimation in generic environments**

We have explained the optic-flow-based attitude estimation with a simplified model, assuming a constant height, direct rate control, a flat floor, and a single optic flow sensor looking straight down from the drone. In the last few sections, we have shown that optic-flow-based attitude estimation is also possible for more complex motions, control, and sloped surfaces. However, an important question is whether it is still possible to estimate attitude in generic environments, with the drone having access to the full optic flow field.

It is well known that if an observer has access to the full flow field, and has a wide enough field of view, it can estimate its unscaled translational velocity, rotation rates, and (inverse) depths to all world points $P_{i}$ in the field of view^5–7^. The “unscaled” pertains to the velocity and inverse depths. Optic flow captures the ratio of velocity and depth, and these quantities cannot be disentangled only based on optic flow itself. Hence, it is not possible to estimate velocity in meters per second and depth in meters. Instead, the velocity vector is typically restricted to have a norm of one, and the inverse depths are scaled accordingly^7^.

Figure ED-3d shows a 2D-case of a drone flying over uneven terrain. From the optic flow field it estimates a unit-vector for velocity $\vec{v}$, with $\left\| \vec{v} \right\|=1$, the rotation rate $p$, and all inverse depths $\frac{1}{z_{Bi}}$ for all world points $P_{i}$ in view^5–7^. We will show below that in such a complex scenario, the drone can use a single feature in its field of view for determining its state.

The main decision to take is how to define $Z_{I}$ in this case. One natural choice would be to take the *height*, i.e., the drone’s distance in the direction of gravity to the closest object. However, in our case the drone does not know the gravity direction, as it needs to estimate its attitude. Hence, we make the choice that $Z_{I}$ is the depth in the gravity direction to a tracked feature $P_{i}$. Hence, the origin of this inertial frame may lie in the air. This choice has as advantage that the drone can track the feature over time (with optic flow), so that in a static environment $Z_{I}$ only changes when the drone has a vertical velocity, i.e., $w_{I}\neq0$. Of course, in this case $Z_{I}=0$ is a “virtual ground plane” and will typically no longer correspond to the actual ground plane. This is not problematic as the drone will have to fly over the obstacles on the terrain, which may hence be more relevant than the actual ground plane. The drone perceives the feature $P_{i}$ under a known angle $\alpha$, which will be part of the observation equations as we will see below.

In this generic case, the observations used by the filter will not be optic flow, but will consist of the rate $p$, and the ratio of the velocity components and estimated depths: $\frac{v_{B}}{Z_{Bi}}$ and $\frac{w_{B}}{Z_{Bi}}$. We use these latter ratios, since the velocities and depths have been determined to make this ratio correct^7^, so they are not expressed in meters per second or meters. For use in the state estimation filter, we need to express these observed ratios in terms of the inertial velocities and $Z_{I}$, leading to:

|  | $\frac{v_{B}}{Z_{Bi}}=\frac{(cos(\varphi)v_{I}-sin(\varphi)w_{I})cos(\varphi+\alpha)}{Z_{I}cos(\alpha)}$ | (Eq. 45) |
| --- | --- | --- |

And:

|  | $\frac{w_{B}}{Z_{Bi}}=\frac{(sin(\varphi)v_{I}+cos(\varphi)w_{I})cos(\varphi+\alpha)}{Z_{I}cos(\alpha)}$ | (Eq. 46) |
| --- | --- | --- |

Please note that due to the definition of the positive rotation in this figure, $\alpha$ is negative in the example in the figure, so that $\varphi+\alpha=\beta$. Using these measurements in nonlinear observability analysis leads to a locally, weakly observable system, without a flat floor assumption. Numerical verification was performed with 10000 samples from state space and $\alpha\in[-60,60]^{\circ}$. It showed that all samples result in a locally, weakly observable state, with an average observability degree of 0.41 and standard deviation 0.12.

The above analysis shows that theoretically, the optic flow field and a single world point suffice for determining attitude, irrespective of environment structure. In practice, relying on a single point would lead to a brittle solution. One solution avenue is to frame the estimation problem in the context of monocular visual Simultaneous Localization And Mapping (SLAM)^8^, and add the gravity vector to the state to be estimated. Another solution avenue is to make the estimation robust by tracking larger groups of features and having $Z_{I}$ represent the average depth to these features in the gravity direction.

In the robotic experiments in this article with the quadrotor we implemented a straightforward scheme to deal with surface irregularities, based directly on the flow field. Instead of measuring the optic flow at a single pixel in the center of the image, we determined $\frac{v_{B}}{Z_{B}}$ by taking the average of the horizontal optic flow. Likewise, we determined $\frac{w_{B}}{Z_{B}}$ by averaging the “size divergence”^9^ of many pairs of points throughout the whole field of view. As a consequence, the estimated $Z_{I}$ coordinate represents the height with respect to the average scene depth.

# **SI-VIII: Simulation verification stability proof**

In SI-II we provide a theoretical proof that the partially unobservable system is attitude stable. In this section, we further clarify and support the proof by means of simulations. The simulations use the state update equation of the constant altitude model with rate control inputs as ground truth dynamics. The drone estimates the state with an Extended Kalman Filter (EKF). The parameters for this (and the following experiments) are the following. The observation covariance matrix assumed by the EKF is $R=0.01$ and the process noise covariance matrix $Q=diag\left( \left[ \begin{matrix} 0.04 & 4^{\circ} & 0.01 \end{matrix} \right] \right)$. The initial covariance matrix $P=diag\left( \left[ \begin{matrix} 1 & 1^{\circ} & 1 \end{matrix} \right] \right)$. The drone uses the current state estimate for the inner loop attitude control to set the rate. The control gain for the inner loop P-controller is $K_{\varphi}=1$. We use an optic flow outer loop controller to set the desired attitude. It is a PI-controller with gains $K_{P}=0.0873$ and $K_{I}=0.00873$. Moreover, we use as desired optic flow $\omega^{*}=0$, i.e., the simulated drone should hover in place. If the flow target is reached, the desired attitude angle will be a constant $\varphi^{*}=0$, implying that the rate will be zero and the state unobservable. We initialize the drone with a velocity $v_{I}=1$ and height $Z_{I}=1$. By default, we initialize the state estimate to be equal to the true state.

**No noise, no disturbances, no observability, no problem**

First, we simulate the situation in which there is no noise and there are no disturbances. There is a small delay of $\Delta t=0.002$s, due to the filtering and control being applied digitally (after simulating the system for a time step $\Delta t$). As stated at the start of part II of the proof, the drone should be able to reach the optic flow reference point and should stay in that desired state, despite it being unobservable.

Figure ED-7a shows the results of this experiment. The true and estimated states overlap, as they are almost equal over the entire run, and the drone reaches the setpoint after ~20 seconds. The error $\omega_{y}-\omega_{y}^{*}$ remains zero when the hover state is reached and the commanded rate converges to zero (Fig. ED-7a, bottom center). The observability degree keeps slowly descending (Fig. ED-7a, bottom right). As explained in the proof, despite the unobservability, the state estimate does not start to wander over the unobservability manifold defined by Eqs. 31 and 33 (SI-II).

**Observation noise**

As our next step, we introduce just observation noise. Specifically, we set $\sigma\left( \omega_{y} \right)=0.02$. This leads to the results shown in Figure ED-7b. Since the optic flow is noisy, it does not converge to the desired flow value. Hence, the controller will never settle at a constant attitude setpoint of $\varphi^{*}=0$. The rate often crosses zero, but never settles to zero (see the zoom in Fig. ED-8a, bottom). Since the outer loop is sensor-based, it directly induces observability. Moreover, we can see that the observation noise leads to differences between the true and estimated state (Fig. ED-8a, top). The estimate is slightly different and delayed with respect to the ground truth. The bottom right plot of Fig. ED-7b shows that the degree of observability oscillates around ~0.17, making the system (slightly) observable.

**Actuation noise**

We now introduce actuation noise instead of sensor noise. Specifically, we add normally distributed noise to the commanded rate, with $\sigma_{p}=1$ ^o^/s. The results of this experiment are very similar to those of introducing sensor noise. The main difference lies in the resulting observability degree over time, shown in Fig. ED-8b. Although in this case the optic flow and hence the rate commands are smooth, the actuation noise itself keeps the rate non-zero, leading to a higher observability varying between ~0.25 and ~0.80.

**Lateral disturbances**

We also introduced lateral disturbances, in the form of lateral accelerations that are normally distributed centered at zero and with standard deviation $\sigma_{a}=0.1$ m/s^2^. Although in this case the optic flow and rate are now smooth, the lateral disturbances accelerate the drone left or right. This results in nonzero optic flow measurements. This has two effects. First, the state estimation filter will attribute the accelerations captured by the measurements to attitude changes, since wind disturbances are not part of the model. This leads to slightly incorrect state estimates. Second, the outer loop controller will react to the changes in optic flow by changing the desired attitude. This results in nonzero rates and hence induces observability. The observability degree (shown in Fig. ED-8c) varies between 0.2 and 0.3.

**Malicious disturbances**

In simulation it is possible to introduce a “malicious” disturbance that only perturbs the system in directions that lie on the unobservable manifold. In the case of the simple model explained in MM-I and studied here, a malicious disturbance is one that perturbs the system in the vertical axis, when the velocity, rate and attitude are all zero. We have implemented such a disturbance that accelerates the system upwards at 1 m/s^2^ when the velocity $v\leq0.01$ m/s. Fig. ED-8d shows the results. Around 25 s the velocity is small enough to apply the vertical acceleration, which is undetected by the filter, since the upward acceleration / velocity is measured by neither the gyro nor the lateral optic flow $\omega_{y}$. Moreover, in this case, there is no other type of disturbance or noise to make the state observable again. Hence, the state change will never be detected.

## **SI-IX: Model with independently moving head and body**

In the majority of the article, we have assumed an elementary quadrotor model. A major feature of the quadrotor model is that the camera is rigidly attached to the body. In contrast, flying insects such as honeybees are known to stabilize their gaze in flight, reducing rotational optic flow. An important question that arises is whether the proposed theory is still valid in such a setup, which has additional degrees of freedom.

Figure ED-10a shows a “honeybee model” in which the body and head can rotate independently of each other, focusing here on the pitch angle. In the model, the thorax and abdomen making up the body are assumed to be a single rigid body. The pitch angle of the body, $\theta_{body}$, determines the wing stroke plane (which is at 90 degrees with the body), and hence the thrust vector direction. The state update equations detailed below only involve the pitch angle of the head $\theta_{head}$ and of the wing stroke plane $\theta_{wings}$, since this latter angle is constant with respect to the body, and it is the wings that produce the thrust. The pitch angle of the head determines the direction of the optic flow sensor, which measures the optic flow for a point on the ground surface at depth $z_{B}$ from the eye. The optic flow sensor can be thought of as a single elementary motion detector that is pointed straight down from the facet eye. The rotation rate of the wing stroke plane and head are independently set, and whereas the former determines the thrust vector direction, the latter influences the perceived optic flow. Finally, the honeybee is aware of the relative angle between its head and body, e.g., by means of neck hairs^10–12^. Hence it also observes the relative angle between the wing stroke plane and the head: $\Delta\theta=\theta_{head}-\theta_{wings}$.

Here we will perform a nonlinear observability analysis for the model under a constant height assumption. The state vector consists of the lateral inertial velocity, head and wing stroke plane pitch angle and the inertial height, i.e., $\vec{\boldsymbol{x}}=\left[ u_{I}, \theta_{head}, \theta_{wings}, z_{I} \right]$. This leads to the following state update equations:

|  | $\left[ \begin{matrix} \dot{u_{I}} \\ \begin{matrix} \dot{\theta}_{head} \\ \dot{\theta}_{wings} \end{matrix} \\ \dot{Z_{I}} \end{matrix} \right]=\left[ \begin{matrix} g tan\left( \theta_{wings} \right) \\ \begin{matrix} q_{head} \\ q_{wings} \end{matrix} \\ 0 \end{matrix} \right]$ | (Eq. 47) |
| --- | --- | --- |

, where the height is constant since the thrust is equal to gravity $g$, leading to a horizontal acceleration $\dot{u_{I}}$ when $\theta_{wings}\neq0$. Moreover, the honeybee controls (and hence knows) the rates of the head and wing stroke plane ($q_{head}$ and $q_{wings}$). Finally, the honeybee model observes both the optic flow $\omega_{x}$ and the relative angle between head and body $\Delta\theta$, leading to the following observation equations:

|  | $\left[ \begin{matrix} \omega_{x} \\ \Delta\theta\end{matrix} \right]=\left[ \begin{matrix} {-u_{I}{cos(\theta_{head})}^{2}}/{z_{I}}+q_{head} \\ \theta_{head}-\theta_{wings} \end{matrix} \right]$ | (Eq. 48) |
| --- | --- | --- |

Combining these measurements with the first two Lie derivatives of $\omega_{x}$ (representing $\dot{\omega_{x}}$ and $\ddot{\omega_{x}}$), leads to an observability matrix $\mathcal{O}$ that is full rank. This means that also this system is locally, weakly observable. If we set either $q_{wings}=0$ or $q_{head}=0$, the observability matrix $\mathcal{O}$ stays full rank. This means that both rotating the wing stroke plane and rotating the head convey information on state changes. Rotating the wing stroke plane induces changes in the sideways acceleration, which can be picked up via the optic flow – similar as to the previously studied quadrotor cases. The information coming from head rotations is related to the angle of the head and (local) flatness of the ground; For example, assuming a positive head pitch angle (looking backwards), a positive head rotation rate $q_{head}$ increases $z_{B}$, reducing $\omega_{x}$. A negative head rotation rate decreases $z_{B}$, increasing $\omega_{x}$. When both $q_{wings}=0$ or $q_{head}=0$, the observability matrix is no longer full rank.

In summary, also a system in which the observation and thrust direction rotate separately is locally, weakly observable. It is important to emphasize that the observability analysis above does not make any assumptions on the specific control scheme for the head or wing stroke plane. It is valid both in the case that the honeybee attempts to keep the head horizontal^13^ or in the case that it lets the eye orientation align with the terrain orientation^14^.

These theoretical observations are confirmed by simulation experiments, implementing the above state update and observation equations. As in other simulations, an EKF receives noisy, delayed optic flow and relative attitude measurements and takes noisy, delayed actions. Fig. ED-10b-g show the results of a single simulation run. In order to have the model better correspond to a robot with flapping wings, a higher drag factor of $k_{0}=3$ is assumed. Moreover, the drag varies with the angle as: $k=cos(\theta)k_{0}$. The control of the head uses the current estimate of the head attitude and attempts to keep it level, i.e., equal to zero degrees, i.e., $\theta_{head}^{*}=0$. To this end, it uses a proportional gain controller on the head attitude error: $p_{head}={-K}_{head}(\hat{\theta}_{head}-\theta_{head}^{*})$. The results of the experiment show that all state variables are well estimated, despite the extra degree of freedom introduced by the independently moving head. Moreover, attitude variation in both the head and body can be seen, also after converging to the right optic flow.

**SI-X: Attitude angle variations at different flight speeds**

**Biological data analysis**

For the results reported on honeybees in Figure ED-2, we performed a novel analysis of the biological data from honeybee experiments by Portelli et al.^15^. In that article, groups of freely flying honeybees (*Apis mellifera*) were color-marked and trained outdoors to enter into a doubly tapered tunnel and fly along it to collect sugar solution at the opposite end. The flight tunnel was 220 cm long, 40 cm high and 25 cm wide at the entrance. The tapering is first vertical, eventually reducing the height to 15 cm, and subsequently horizontal, reducing the width to 5 cm. After that, the tunnel became wider again. As a consequence of the tapering, the honeybees flew at different speeds in different parts of the tunnel, which was the most important reason for analyzing this data. Moreover, the honeybees’ trajectories were filmed at a rate of 20 frames per second (Ts= 50 ms) with a high-resolution digital black-and-white CMOS camera. The resulting images were processed with custom MATLAB scripts to extract the positions, velocities and pitch attitudes over time. The pitch was extracted from the main orientation of an ellipse fit of the dark bee patterns automatically detected in the images. For additional details, please refer to Portelli et al.^15^.

For the analysis in this article, we first accumulated the pitch angle data from all 21 honeybee trajectories in eight velocity bins, i.e., [0.05, 0.15) cm/s, [0.15, 0.25) cm/s, …, [0.75, 0.85) cm/s, where “)” means that the interval maximum itself is excluded. Subsequently, outlier pitch angles were removed by the standard “rmoutlier” MATLAB function. This function removes 22 out of the total of 1994 samples. The standard deviations of the resulting pitch sample distributions are shown in the plots in Fig. ED-2. Moreover, Fig. ED-9 shows the histograms of pitch angles for the various velocity bins with and without the outliers in the data.

**Simulation experiments**

We compare the trend in the biological data with simulation results, with the model from SI-IX, in which the head and body can rotate independently. As explained in SI-IX, the head control has as objective to stay level (pitch attitude zero), while the body angle is controlled to achieve a given desired longitudinal ventral optic flow. For the experiments, we ran N=30 simulations per desired velocity. Each desired velocity is located in the center of the velocity intervals used for the honeybees. The constant height, body-head model was used, employing both lateral flow $\omega_{x}$ and divergence $\omega_{z}$ and taking into account drag (with a drag factor of $k_{d}=2.0$). Optic flow was measured at 30 Hz in simulation, with a standard deviation of normally distributed added noise of $\sigma_{\omega}=0.02$ and similarly distributed rotation rate actuation noise of $\sigma_{q}=0.005$. Furthermore, commanded rates were delayed with 0.01 s before having effect in the simulator. The simulator starts in a hover configuration at a height z = 1m, which remains fixed due to the constant height nature of the model. The simulated drone has a desired optic flow value of $\omega_{x}^{*}=v_{x}^{*}$, since z = 1m. The simulation runs for 30 seconds. The data used to evaluate the standard deviation of the roll angle comes from the second half of the simulation, [15,30] s, to ensure that the simulated drone has converged to the setpoint, and that any oscillations in attitude are not due to transient control effects in response to offsets between the initial and desired state, but due to persisting issues at the desired flight speed. All attitude angles are aggregated over the N simulations per condition, and the standard deviation is taken, resulting in the $\sigma_{\theta}$ values shown in Figure ED-2. Given that in simulation we have access to ground truth state values, we can also determine the errors of the state estimator for the different velocities.

We have also performed simulation experiments with a model that does *not* use the proposed estimator, but attitude estimates that are noisy versions of the ground truth: $\hat{\theta}=\theta+\varepsilon$, with the noise distributed normally, i.e., $\varepsilon\sim\mathcal{N}\left( 0,\sigma_{\varepsilon} \right)$ with $\sigma_{\varepsilon}=0.25$. In contrast to the proposed optic-flow-based estimator, the accuracy of the estimates $\hat{\theta}$ of this (further identical) version of the simulation model does not depend on the state, and hence also not on velocity. It allows us to piece apart aerodynamic drag effects from observability effects.

**Results**

Here, we briefly discuss the results in Figure ED-2. The honeybees in the experiments have an attitude and velocity that vary over time. Figure ED-2b,c show a representative trajectory over time. The standard deviation of the pitch attitude averaged over the 21 trajectories is $11.39^{\circ}$. The faster the honeybees fly, the lower their pitch angle becomes (Figure ED-2d). In the coordinate frame of the biological experiments, this means that they fly more horizontally. This is in accordance with a thrust-vectoring model.

A main observation mentioned in the discussion of the main article is that the attitude variation of the honeybees decreases for increasing flight speeds (Figure ED-2e). The simulation models show a similar trend to the biological data, albeit with a different magnitude. Interestingly, both models show a reduction in body attitude variation for higher flight speeds, which in our simulator is due to the parasitic drag model. However, the model with the optic-flow-based state estimator has a higher body attitude variation at lower speeds, and a slightly lower variation at higher speeds. This suggests that additional effects to aerodynamic drag play a role. Remarkably, the difference between the two models is substantial when it comes to the attitude variation of the head (Figure ED-2f); The optic-flow-based estimator results in much higher head attitude variation than the noisy ground-truth estimator. Moreover, only the optic-flow-based estimator shows the trend of reducing attitude variation at higher flight speeds. This clear difference can be explained by the theoretical analysis, which shows that the observability of the state is higher for higher flight speeds (Figure ED-2g). Indeed, the mean absolute state estimation errors decrease with increasing flight speed (Figure ED-2h).

# **References supplementary material**

1. Kou, S. R., Elliott, D. L. & Tarn, T. J. Observability of nonlinear systems. *Inf. Control* **22**, 89–99 (1973).

2. Hermann, R. & Krener, A. Nonlinear controllability and observability. *IEEE Trans. Automat. Contr.* **22**, 728–740 (1977).

3. Krener, A. J. & Ide, K. Measures of unobservability. in *Proceedings of the 48h IEEE Conference on Decision and Control (CDC) held jointly with 2009 28th Chinese Control Conference* 6401–6406 (2009).

4. de Croon, G. C. H. E. *et al.* Optic-flow based slope estimation for autonomous landing. in *IMAV 2013* (2013).

5. Longuet-Higgins, H. C. & Prazdny, K. The interpretation of a moving retinal image. *Proc. R. Soc. London, B Biol. Sci.* **208**, 385–397 (1980).

6. Koenderink, J. J. & van Doorn, A. J. Facts on optic flow. *Biol. Cybern.* **56**, 247–254 (1987).

7. Jaegle, A., Phillips, S. & Daniilidis, K. Fast, robust, continuous monocular egomotion computation. in *2016 IEEE International Conference on Robotics and Automation (ICRA)* 773–780 (2016).

8. Fuentes-Pacheco, J., Ruiz-Ascencio, J. & Rendón-Mancha, J. M. Visual simultaneous localization and mapping: a survey. *Artif. Intell. Rev.* **43**, 55–81 (2015).

9. De Croon, G. C. H. E. Monocular distance estimation with optical flow maneuvers and efference copies: a stability-based strategy. *Bioinspir. Biomim.* **11**, 1–18 (2016).

10. Wilson, M. The functional organisation of locust ocelli. *J. Comp. Physiol.* **124**, 297–316 (1978).

11. Srinivasan, M. V. Honeybees as a model for the study of visually guided flight, navigation, and biologically inspired robotics. *Physiol. Rev.* **91**, 413–460 (2011).

12. Preuss, T. & Hengstenberg, R. Structure and kinematics of the prosternal organs and their influence on head position in the blowfly Calliphora erythrocephala Meig. *J. Comp. Physiol. A* **171**, 483–493 (1992).

13. Boeddeker, N. & Hemmi, J. M. Visual gaze control during peering flight manoeuvres in honeybees. *Proc. R. Soc. B Biol. Sci.* **277**, 1209–1217 (2010).

14. Expert, F. & Ruffier, F. Flying over uneven moving terrain based on optic-flow cues without any need for reference frames or accelerometers. *Bioinspir. Biomim.* **10**, 26003 (2015).

15. Portelli, G., Ruffier, F., Roubieu, F. L. & Franceschini, N. Honeybees’ speed depends on dorsal as well as lateral, ventral and frontal optic flows. *PLoS One* **6**, e19486 (2011).

Supplementary Videos – supplied separately

Supplementary video 1:

**Quadrotor flying with optic-flow-based attitude.**

A Parrot Bebop 2 quadrotor’s onboard software has been reprogrammed with the Paparazzi open-source autopilot in order to hover fully autonomously. Initially, it flies with an inner loop control for the attitude that is based on a standard, complementary attitude estimation filter. This filter combines accelerometer with gyro measurements. During this initial part of the experiment the outer loop control already relies on optic flow. The optic flow divergence is used to set thrust commands, the translational optic flow is used to set attitude commands, executed by the inner loop control. Halfway the experiment, the attitude estimation is switched to the proposed optic-flow-based attitude estimation. This estimation combines a constant-height, thrust-vectoring motion model with optic flow and gyro measurements to estimate both pitch and roll angles.

Supplementary video 2:

**Flapper drone flying with optic-flow-based attitude.**

A “Flapper drone” flapping wing robot has been equipped with an artificial compound eye, “CurvACE”. The autopilot software of the Flapper drone, based on BitCraze’s CrazyFlie code, has been modified to implement the proposed optic-flow-based attitude estimation. The CurvACE has been programmed to send translational optic flow measurements to the autopilot at 200 Hz. At the start of the experiment, the inner loop attitude control relies on a standard complementary attitude estimation filter, combining accelerometer and gyro measurements. The outer loop control in this experiment consists of manual control, with the human pilot commanding desired attitude angles for use by the inner loop controller. After a bit of flight, a switch is made to the proposed optic-flow-based attitude estimation, only for the roll angle in this experiment. The optic-flow-based attitude estimation combines a constant, thrust-vectoring motion model with CurvACE’s optic flow measurements and gyros.

Supplementary video 3:

**Quadrotor flying over a tilted slope.**

The video shows an experiment to verify the robustness of the proposed optic-flow-based attitude estimation to tilted surfaces. A Parrot Bebop 2 drone with onboard Paparazzi open-source autopilot flies over a tilted slope. The autopilot features an optic flow outer loop control with optic flow divergence leading to thrust commands and translational optic flow to attitude commands. The outer loop control attempts to achieve zero optic flow, i.e., hover flight. Initially, the inner loop attitude control uses attitude estimates from a standard complementary filter. After a bit of flight, the attitude estimation is changed to the proposed optic-flow-based attitude estimation, combining a motion model with optic flow and gyro measurements to estimate both pitch and roll. The motion model is based on a flat floor assumption. In this experiment, that assumption is violated, as the drone flies over a tilted screen. In the experiment, first the screen’s tilt angle is increased. Then, the screen is dragged to the side.

Supplementary video 4:

**Quadrotor flying over 3D structure.**

This experiment tests the robustness of the proposed optic-flow-based attitude estimation to non-flat surfaces. A Parrot Bebop 2 drone with onboard the open-source autopilot Paparazzi flies over an area with differently sized plastic plants and chairs. The autopilot features an optic flow outer loop control with optic flow divergence leading to thrust commands and translational optic flow to attitude commands. The optic flow observables, i.e., divergence and translational flow, are determined by integrating information from the optic flow vectors in the entire bottom camera field of view. The outer loop control follows different optic flow set points over the experiment, starting with zero flow for hover flight. The attitude estimation starts out with a standard complementary filter, and then switches to the proposed optic-flow-based attitude estimation, combining a motion model with optic flow and gyro measurements to estimate both pitch and roll. The motion model is based on a flat floor assumption. In this experiment, that assumption is violated by means of the objects in the flight arena. The drone first hovers over a large plastic plant, with the leaves moving due to the downwash, violating an additional assumption of a static world. Then the drone receives different outer loop references for nonzero translational optic flow, making it move left and right over the three-dimensional scene.

Supplementary video 5:

**Quadrotor subjected to 10-degree roll disturbances.**

This experiment tests the robustness of the proposed optic-flow-based attitude estimation to large disturbances. A Parrot Bebop 2 drone with onboard open-source autopilot Paparazzi flies with optic flow outer loop control, with optic flow divergence leading to thrust commands and translational optic flow to attitude commands. The outer loop has zero divergence and lateral flow as optic flow references, for hover flight. Initially, the attitude is estimated with a standard complementary attitude estimation filter. Then, the drone switches to the proposed optic-flow-based attitude estimation scheme for both pitch and roll and first hovers. At multiple times in the video, the experimenter introduces a disturbance to the roll angle by extraneously adding a bias to the attitude command sent to the inner loop attitude controller. So, the drone is hovering and sending a desired angle of zero degrees to the inner loop attitude controller, but this is extraneously changed to 10 degrees. The inner loop controller attempts to satisfy this demand, resulting in an increasing sideward velocity. The situation is corrected for by the integrator in the outer loop controller, which attempts to cancel the velocity by changing the desired attitude angle, i.e., -10 degrees when hovering again. Then, the experimenter introduces a new disturbance by removing the bias, resulting in the opposite motion. Mats were placed on the floor to ensure that the optic flow algorithms functioned correctly also at higher speeds.

Supplementary video 6:

**Quadrotor flying over a static slope - varying height model.**

The video shows an experiment to verify the robustness of the proposed optic-flow-based attitude estimation to tilted surfaces. A Parrot Bebop 2 drone with onboard Paparazzi open-source autopilot flies over a tilted slope. The autopilot features an optic flow outer loop control with optic flow divergence leading to thrust commands and translational optic flow to attitude commands. The outer loop control attempts to achieve zero optic flow, i.e., hover flight. Initially, the inner loop attitude control uses attitude estimates from a standard complementary filter. After a bit of flight, the roll angle attitude estimation is changed to the proposed optic-flow-based attitude estimation, combining a motion model with optic flow and gyro measurements. In contrast to previous experiments, the motion model does not assume a constant height. The model uses the estimated commanded thrust in order to predict height changes. The motion model does still assume a flat floor. In this experiment, that assumption is violated, as the drone flies over a tilted screen. In the experiment, the screen’s tilt angle is increased.

Supplementary video 7:

**Quadrotor flying over a moving slope - varying height model.**

The video shows an experiment to verify the robustness of the proposed optic-flow-based attitude estimation to moving, tilted surfaces. A Parrot Bebop 2 drone with onboard Paparazzi open-source autopilot flies over a tilted slope. The autopilot features an optic flow outer loop control with optic flow divergence leading to thrust commands and translational optic flow to attitude commands. The outer loop control attempts to achieve zero optic flow, i.e., hover flight. Initially, the inner loop attitude control uses attitude estimates from a standard complementary filter. After a bit of flight, the roll angle attitude estimation is changed to the proposed optic-flow-based attitude estimation, combining a motion model with optic flow and gyro measurements. The motion model uses the estimated commanded thrust in order to predict height changes. The motion model does still assume a flat floor. In this experiment, that assumption is violated, as the drone flies over a tilted screen. In the experiment, first the screen’s tilt angle is increased. Then, the screen is dragged to the side, with the drone following.

Supplementary video 8:

**Quadrotor flying over 3D structure – varying height model.**

This experiment tests the robustness of the proposed optic-flow-based attitude estimation to non-flat surfaces when the motion model allows for varying height. A Parrot Bebop 2 drone with onboard the open-source autopilot Paparazzi flies over an area with differently sized plastic plants, flowers, boxes and a chair. The autopilot features an optic flow outer loop control with optic flow divergence leading to thrust commands and translational optic flow to attitude commands. The optic flow observables, i.e., divergence and translational flow, are determined by integrating information from the optic flow vectors in the entire bottom camera field of view. The outer loop control follows different optic flow set points over the experiment, starting with zero flow for hover flight. The attitude estimation starts out with a standard complementary filter, and then switches to the proposed optic-flow-based attitude estimation, combining a motion model with optic flow and gyro measurements to estimate roll. The motion model uses the estimated commanded thrust in order to predict height changes and is based on a flat floor assumption. In this experiment, that assumption is violated by means of the objects in the flight arena. The drone first hovers over the center of the scene (the chair). Then the drone receives different outer loop references for nonzero translational optic flow, making it move left and right over the three-dimensional scene.
